# Factors influencing appropriate vestibular care: An interview study with general practitioners and patients

**DOI:** 10.1080/13814788.2025.2600144

**Published:** 2025-12-16

**Authors:** Hà T.N. Ngo, Otto R. Maarsingh, Pauline Slottje, Marco H. Blanker, Jettie Bont, Vincent A. van Vugt

**Affiliations:** ^a^ Department of General Practice, Amsterdam UMC, Location Vrije Universiteit Amsterdam, Amsterdam, The Netherlands; ^b^ Amsterdam Public Health research institute, Amsterdam, The Netherlands; ^c^ Department of Primary and Long-term care, University Medical Centre Groningen, Groningen, The Netherlands; ^d^ Department of General Practice, Amsterdam UMC, location AMC, Amsterdam, The Netherlands

**Keywords:** Primary health care, general practice, vestibular disorders, dizziness, vertigo, qualitative research

## Abstract

**Background:**

General practitioners (GPs) frequently prescribe anti-vertigo drugs (AVDs), even though there is limited evidence for their effectiveness. Meanwhile, they rarely apply vestibular rehabilitation, a treatment for various vestibular disorders with a strong evidence base.

**Objectives:**

This study aimed to identify barriers and facilitators to appropriate vestibular care in general practice.

**Methods:**

We conducted a qualitative study in Dutch general practice using semi-structured interviews with GPs and patients with vestibular symptoms. We used purposive sampling to select participants. Interviews were audio-recorded, transcribed verbatim, and thematically analysed following the Template Analysis approach using MAXQDA 2022 software.

**Results:**

We interviewed 11 GPs and 15 patients. We assessed barriers and facilitators to appropriate vestibular care for GPs (i.e. not prescribing AVDs, advising vestibular exercises) and patients (i.e. not using AVDs, doing vestibular exercises). We identified four themes: competence, mindset, relational determinants, and accessibility to care. Facilitators included adequate knowledge about vestibular disorders, GPs valuing delivering high-value care, positive experiences with physiotherapy, patients’ coping skills, personal continuity, close collaboration between GP and colleagues, social support, and sufficient time and availability of providers. Barriers included diagnosis and treatment insecurity among GPs, patients doubting the GPs’ competence, patients’ desperation for treatment and GPs accommodating these wishes, positive experiences with AVDs, prescriptions by other providers, and insurance not covering physiotherapy.

**Conclusion:**

Multiple barriers and facilitators shape appropriate vestibular care in general practice. Interventions should strengthen GPs’ and patients’ knowledge of vestibular management. Internet-based vestibular rehabilitation may address key barriers, particularly logistic and financial ones.

## KEY MESSAGES

- This interview study combines general practitioners’ and patients’ perspectives on managing vestibular symptoms.
- We focused on identifying targets to improve vestibular care by reducing overprescription of anti-vertigo drugs and increasing the uptake of vestibular rehabilitation.
- Key targets include increasing knowledge of general practitioner and patient, and providing internet-based vestibular rehabilitation.

## Introduction

Vestibular symptoms, such as dizziness and vertigo, account for up to 16% of general practitioner (GP) consultations [1]. Peripheral vestibular disorders such as benign paroxysmal positional vertigo (BPPV), vestibular neuritis, and Ménière’s disease are among the most common causes of vestibular symptoms, with prevalences of 14–34% [1,2]. Persistent postural-perceptual dizziness (PPPD) is another increasingly recognised cause of chronic vestibular symptoms, often developing after an acute vestibular disorder and contributing substantially to long-term impairment in primary care [3]. Vestibular disorders often coexist with anxiety and depressive symptoms, which can exacerbate disability and delay recovery; therefore, accurate diagnosis and targeted treatment are essential [4].

Depending on the underlying cause, guidelines recommend specific evidence-based treatments. One of the evidence-supported and widely applicable approaches is vestibular rehabilitation (VR), an exercise-based treatment aimed at restoring balance function through repeated eye, head, and body movement exercises that promote central compensation. Typical components include gaze stabilisation, balance, and habituation exercises, which can be delivered by physiotherapists or through online self-training programs [5–7]. VR is recommended for unilateral vestibular hypofunction (e.g. vestibular neuritis), bilateral vestibular hypofunction (e.g. due to ototoxicity), and chronic vestibular symptoms regardless of the initial aetiology.

Despite this strong evidence base, only 6–7% of GPs apply VR, e.g. by referring patients to physiotherapists, according to surveys among Dutch and British GPs [8,9]. In contrast, GPs worldwide frequently prescribe anti-vertigo drugs (AVDs), such as betahistine [10–12], even though there is limited evidence supporting their effectiveness. There is ongoing discussion about the role of AVDs, particularly for patients with Ménière’s disease, and recommendations vary between countries. Betahistine has no FDA approval for any vestibular disorder in the United States. In the Netherlands and the United Kingdom, it is approved only for Ménière’s disease but is also frequently prescribed for other vestibular disorders [13,14]. Despite this off-label practice, large randomised controlled trials, such as the BEMED study [15], and recent Cochrane reviews have found no clinically meaningful benefit of betahistine for vestibular outcomes, and the overall certainty of the evidence remains low [16,17]. Adverse effects are generally mild—mostly gastrointestinal discomfort and headache—but the current evidence does not support its routine use for other indications than Ménière’s disease. Therefore, betahistine is not recommended as a general anti-vertigo medication, and prescriptions for other vestibular disorders, especially for long-term use, can be considered inappropriate. In accordance with this, Dutch guidelines have advised against these drugs for decades [18], but Dutch GPs still prescribe these drugs in nearly 1 in 10 patients with vestibular symptoms [19].

These findings show that GPs’ adherence to guideline-recommended treatments needs to be improved. Improving vestibular care is essential, as these symptoms markedly reduce quality of life, limit daily functioning, and impose a substantial economic burden through sick leave and frequent healthcare visits [20,21]. In order to enhance appropriate vestibular care, we need to understand what barriers and facilitators play a role. For that reason, we carried out this qualitative interview study.

## Methods

### Study design

We conducted semi-structured interviews with GPs and patients with vestibular symptoms to explore barriers and facilitators to appropriate vestibular care in general practice. Specifically, we evaluated barriers and facilitators to prescribing/using AVDs and to advising/doing exercises that improve vestibular function, such as VR. We adopted a realist approach to thematic analysis in this study, assuming that phenomena have a real, objective existence [22]. We used the Template Analysis, in which a coding template is developed and a priori themes may be defined. Template Analysis is not bound to a specific philosophical stance, allowing emphasis on data reliability and transparency [23,24]. We reported this study using the COREQ checklist [25]. The medical ethical committee of the VU University Medical Centre approved the study protocol on 27 June 2022 (protocol 2022.0436).

### Participants and recruitment

We recruited GPs, seeking variety in age, gender, years of practice, location of practice (urban/rural and region), size of practice (number of employed GPs and registered patients), and self-reported prescription behaviour regarding AVDs. GPs were invited through the academic general practice network of Amsterdam UMC (ANHA) (newsletter and annual meeting) and the research team’s professional network *via* direct and online contact. Patients aged ≥18 with current or past vestibular symptoms were recruited through participating GPs and the patient association (Stichting Hoormij•NVVS), which shared our invitation on Facebook. Inclusion criteria were adults with vestibular symptoms of any cause. We included patients with BPPV, vestibular neuritis, Ménière’s disease, vestibular migraine, and PPPD. Exclusion criteria were inability to provide informed consent, insufficient Dutch language proficiency, or acute systemic, neurological, or psychiatric conditions unrelated to vestibular disorders. No vestibular disorder types were excluded to reflect general-practice diversity. We sought variation in demographics, level of education [26], diagnosed vestibular disorder, and AVD use. Interviews were scheduled shortly after selection, and written consent was obtained. Interviews continued until the team agreed data were sufficient to address the research questions [27].

### Interviews

We developed separate topic lists for GPs and patients, using our own clinical and academic observations, literature, and the Theoretical Domains Framework (Supplementary tables 1 and 2) [28,29]. The interviewer independently revisited the topic list during each interview and added topics when relevant. Interviews were conducted between September 2022 and January 2023 by HTNN at the general practice, patients’ homes, or online (Microsoft Teams). Field notes were written immediately after each interview with thoughts and comments to reflect on during the analysis. All interviews were audio-recorded, transcribed verbatim, anonymised and approved by participants (member check). Transcripts were stored digitally for 15 years.

### Analysis

We followed Template Analysis. Data analysis started after most interviews had occurred and then took place concurrently. Two researchers (HTNN, VAvV) independently coded transcripts, compared codes, reflected on them, developed an initial coding template, and discussed preliminary themes. The template was iteratively refined until final themes and their relationships were agreed upon with the research team. Interviews were analysed in Dutch using MAXQDA 2022 (VERBI Software), and translated quotes were checked by the authors.

### Research team and reflexivity

At the time of the interviews, HTNN (female GP resident/PhD student) had limited prior experience and approached the topic with curiosity. She did not know any patients. Beforehand, she completed two qualitative-research courses. VVaV (male GP/postdoctoral researcher) had prior qualitative experience and a more critical view, but remained aware of his perspective. ORM, MHB, and JB were senior GP researchers; PS was research coordinator at ANHA. Some participating GPs had professional connections to the team, but all data were anonymised and non-traceable.

## Results

### Participant characteristics

We conducted interviews with 11 GPs and 15 patients. During one interview (GP4), the audio recording failed, but a summary of the interview was written down afterwards and sent for member check so that we could reflect on her perspectives during data analysis.

The GPs’ age ranged from 31 to 62 years old, 81% was female, and 46% was not opposed to prescribing AVDs. Practice size ranged from 2 to 5 GPs (1.500–12.000 registered patients), and 73% of the GPs worked in a urban practice. Patients’ age ranged from 41 to 71 years old, 87% was female, 40% had a high level of education, 67% lived in a urban area, 27% currently used AVDs, and 53% currently did vestibular exercises. The majority of patients (73%) had the diagnosis PPPD. BPPV was diagnosed in 33% of patients, and less common were the diagnoses vestibular neuritis (13%), Ménière’s disease (13%), and vestibular migraine (7%). Most patients (86%) had had vestibular symptoms for over a year. Among patients who reported current or past AVD use, the most frequently mentioned drugs were betahistine and cinnarizine. Table 1 shows the participants’ characteristics.

**Table 1.** Characteristics of interviewed general practitioners and patients.

	General practitioners (*n* = 11)^1^		Patients with vestibular symptoms (*n* = 15)
Age in years, mean (range)	44 (31–62)	Age in years, mean (range)	57 (41–71)
Gender, *n* (%)		Gender, *n* (%)	
Female	9 (81)	Female	13 (87)
Male	2 (19)	Male	2 (13)
Years of practice, mean (range)	11 (1–28)	Level of education^b^, *n* (%)	
		Low/secondary	9 (60)
		High	5 (40)
Location practice^c^, *n* (%)		Place of residence^c^, *n* (%)	
Urban	8 (73)	Urban	10 (67)
Rural	3 (27)	Rural	5 (33)
Number of employed GPs at practice, mean (range)	3 (2–5)	Diagnosed vestibular disorder^d^, *n* (%)	
		BPPV	5 (33)
		Vestibular neuritis	2 (13)
		Ménière’s disease	2 (13)
		Vestibular migraine	1 (7)
		PPPD	11 (73)
		Other	2 (13)
Number of registered patients at practice, mean (range)	4580 (1500–12000)	Duration of complaints, *n* (%)	
		0–1 years	2 (13)
		2–5 years	5 (33)
		>5 years	8 (53)
Prescribes anti-vertigo drugs, *n* (%)		Uses anti-vertigo drugs, *n* (%)	
Yes	5 (46)	Yes	4 (27)
No	3 (27)	No	8 (53)
Not anymore	3 (27)	Not anymore	3 (20)
		Does vestibular exercises, *n* (%)	
		Yes	8 (53)
		No	5 (33)
		Not anymore	2 (13)

*BPPV* = benign paroxysmal positional vertigo, *GP* = general practitioner, *PPPD =* Persistent Postural-Perceptual Dizziness.

^a^In one interview with general practitioner number 4 the audio recording failed, we could therefore only use a written summary to reflect on during the analysis.

^b^Low/secondary: primary education, special needs primary education, prevocational secondary education, secondary vocational education level 1 or equivalent, first three years of senior general secondary education or pre-university secondary education, senior years of senior general secondary education or pre-university secondary education, secondary vocational education levels 2, 3 or 4. High: higher vocational education, university bachelor’s or master’s degree, PhD.

^c^Urban: >1.500 surrounding addresses. Rural: <1.500 surrounding addresses.

^d^Some patients reported multiple vestibular disorders.

### Barriers and facilitators to appropriate vestibular care

**Figure 1.**
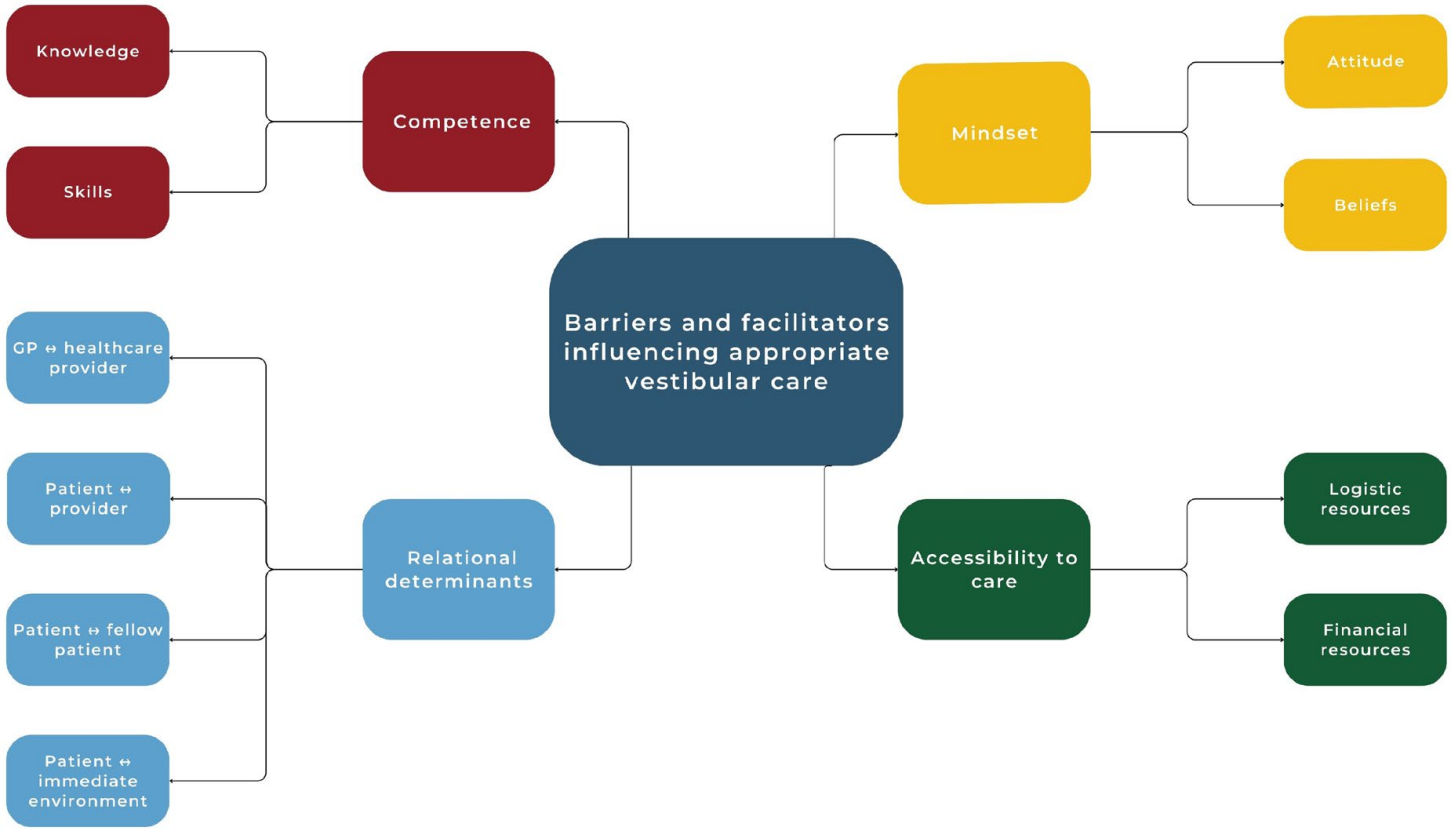
Graphical presentation of barriers and facilitators influencing appropriate vestibular care.

We identified four themes that comprised both barriers and facilitators to appropriate vestibular care for GPs (i.e. not prescribing AVDs, advising vestibular exercises) and patients (i.e. not using AVDs, doing vestibular exercises). The main themes (Competence, Mindset, Relational Determinants, and Accessibility to Care) are depicted in Figure 1. Supplementary figures 1–4 detail each theme with subthemes.

#### Competence

The competence included the knowledge of GPs and patients regarding (the management of) vestibular disorders, and the skills of GPs regarding the diagnosis of vestibular disorders.

##### Facilitators to appropriate care

For GPs, their knowledge determined what treatments they employed. Some GPs had learned that AVDs were not evidence-based, which acted as a barrier to prescribing AVDs. GP7: ‘*It was stressed [during residency] that it is not effective and that it has side effects. So it is not a habit to prescribe them*.’ Having knowledge about the competencies of the local physiotherapist facilitated referral for exercises. GP11: ‘*If I had someone like that at 10 min distance from my practice, I would call them and ask: ‘I have a patient – would it be an idea to refer that patient to you?’, and ask them ‘What kind of things do you do and when is it useful or not to refer patients?’. That way, I would get a better sense of their work.’*

For patients, knowledge could also be a barrier to AVD use. Some knew that medication existed, but that it was not effective for them. P10: ‘*Only people with Ménière’s use those drugs, I think betahistine or cinnarizine, but they don’t work for PPPD.’* Knowledge in patients also played a role when it came to doing exercises. P6: *‘When the symptoms gets worse, I can resume the exercises again and then I know that I can manage them a bit.’*

##### Barriers to appropriate care

Some GPs were insecure about their skills to diagnose and treat patients with vestibular symptoms. These insecurities could facilitate AVD prescriptions and/or be a barrier to advising exercises. GP11: ‘*I once saw a young woman, and I was not sure if it was BPPV or something else. So I wondered: is it wise to do the Epley, or would that only make it worse.’* GPs mentioned that these insecurities were brought on by suboptimal education and having too little exposure to patients with vestibular symptoms.

For patients, lacking knowledge about (better) treatment options could deter them from receiving appropriate care. P9: ‘*I can’t think of any specific treatments for dizziness, apart from benzodiazepines and all that other garbage.’*

#### Mindset

The mindset consisted of the GPs’ and patients’ attitudes and beliefs.

##### Facilitators to appropriate care

For some GPs, delivering high-value care, i.e. cost-conscious and evidence-based care, was important and this acted as a barrier to prescribing AVDs. GP8: ‘*Our job is to deliver cost-conscious care. If medication is not useful, but patients still take it, then it is an unnecessary expense.’* Furthermore, a positive attitude towards (the effect of) exercises and/or physiotherapy facilitated referral. GP8: ‘*I tend to be physiotherapy-minded, I would refer almost anyone if I think they could benefit. I trust the physiotherapist to decide what is helpful or not.’*

Similarly, for patients, a positive view on doing exercises and/or physiotherapy prompted them to do those exercises, while having a more critical view on medication acted as a barrier to using AVDs. P3: ‘*Everything I hear is that there are many side effects to every treatment, so I just think: well, better not do that.’* Lastly, different coping strategies could facilitate appropriate care. Some patients accepted the complaints, which acted as a barrier to using AVDs. Other patients felt desperate to try every treatment in the hope that their symptoms would decrease, which could encourage them to do exercises.

##### Barriers to appropriate care

However, those feelings of desperation in the patient could also facilitate AVD use. P1: ‘*I could not do anything. I sat at home in a chair and every movement that my husband made bothered me. I could not walk or even stand my grandchildren. So I sat there thinking: ‘I have to do something’. That is when you start trying everything.’* Having a positive attitude towards AVDs also facilitated their use. P13: ‘*The symptoms are always present on the background, they are suppressed a bit, but they are there, and I actually think that I will use AVDs until I die.’* On the other hand, a negative attitude towards exercises, for instance because of prior experiences, acted as a barrier to doing exercises. P7: ‘*It does not feel safe to do the exercises. The physiotherapist suggested: ‘I will stay here and do the exercises with you’. A friend of mine said the same thing, but I know that these exercises can be pretty intense.’*

Numerous GPs responded to aforementioned desperation of patients by accommodating their wishes. This facilitated AVD prescriptions. GP8: ‘*I know that the effect is minimal, but I am not taking it away from her. She believes in it, she says that it helps her.’* Some GPs believed that stopping AVDs could increase stress levels in the patient, which also facilitated prescriptions. Lastly, a negative attitude towards physiotherapy, due to prior negative experiences and/or lack of positive experiences, deterred GPs from referring. GP7: ‘*I have not yet found a physiotherapist who makes me think: ‘He or she will tackle this’. If you hear success stories, you might think: ‘Oh, I should really try that’, but now…’*

#### Relational determinants

Relational determinants encompassed GPs’ relationships with other healthcare providers, patient-provider relationships, and patients’ relationships with fellow patients and their immediate environment.

##### Facilitators to appropriate care

For GPs, personal continuity could act as a barrier to prescribing AVDs. GP8: ‘*You can’t just say: ‘Let’s stop this [medication]’. You have to discuss it with them. But they do accept it, if you explain why. I have patients who have been with the practice for 17 years, so they trust me when I say: ‘I think it is good to do this or that’.’* Another barrier to prescribing AVDs was a close collaboration with colleagues, which stimulated GPs to be critical of their own actions. GP1: ‘*I am also a GP trainer, so dizziness is always on the agenda during our learning conversations. So a GP resident can call you out, saying: ‘Hey, why are you still prescribing this?’’*

For patients, receiving support from fellow patients could act as a barrier to using AVDs, as it helped them cope with their complaints. Patients also mentioned that receiving support from their immediate environment (e.g. their partner) was essential to receive care, for instance because of a reduced mobility. P6: ‘*It is insane, but he [partner] has brought me everywhere the last three years.’* Lastly, patients mentioned that they valued clear communication and a clear treatment strategy from their healthcare provider: they argued that this gave them a feeling of reassurance and therefore kept them from looking for other treatments.

##### Barriers to appropriate care

Some GPs felt the need to develop a good relationship with the patient, which could prompt AVD prescriptions. GP11: *‘I think: ‘I am a young doctor, people don’t know me that well yet, they have to learn to trust me as a GP’. So if they really want it [AVDs], I figure giving it to them will help me gain trust for next time.’* Prescriptions for AVDs by other healthcare providers, like ENT specialists, also facilitated prescriptions by GPs.

For patients, doubts about the (capabilities of the) healthcare provider could act as a facilitator to AVD use, since it prompted them to search for reassurance and/or possibilities. P12: ‘*They don’t have the knowledge about what I have, so for them, I am the expert. Every treatment that I tried, they heard about it through me.’ [Interviewer: ‘So the GP didn’t suggest any treatments, except for a referral?’] ‘Just a referral, because I was already looking for treatments myself, and because they didn’t have the knowledge.’* Additionally, hearing positive experiences from fellow patients with AVDs facilitated use of these drugs. P1: ‘*When I see someone with the same disorder and they say: ‘I use betahistine’, I think: ‘Oh I should look into that too’’* Lastly, being alone or feeling lonely acted as a barrier to doing exercises for patients. P7: ‘*I really dreaded the exercises, even though I knew they worked. I did them for a while when my husband was still alive, and the complaints were much better then. But since he passed away, I definitely have not been doing them over the last three years.’*

#### Accessibility to care

Accessibility to care included logistic and financial resources, which influenced both GPs and patients.

##### Facilitators to appropriate care

For GPs, having a physiotherapist in the vicinity of the general practice made it easier to refer patients for exercises. Furthermore, GPs mentioned that insurance not covering AVDs acted as a barrier to prescriptions. GP2: ‘*Insurance has not covered cinnarizine for a few years now. There was a time it was covered, so back then people wanted it. But if you tell them it does not work well and that they have to pay for it themselves, they will quickly say: ‘No’’.*

##### Barriers to appropriate care

In the Netherlands, vestibular physiotherapy is often only partially covered by health insurance, which GPs identified as a barrier to care. GP7: ‘*I work in a disadvantaged neighbourhood, and many people don’t have additional insurance for physiotherapy. That group is harder to help, so I try to help them with exercises myself.’*

Similarly, for patients, not being able to pay for treatments was mentioned as a barrier to doing exercises. P2: *‘You have to pay for it yourself, which means giving up part of your salary to cover treatments, but eventually, the money runs out.’* This situation may differ across countries, but it highlights the importance of financial coverage as a key factor in implementation. The availability of a vestibular physiotherapist in the vicinity also played a role. P11: ‘*I have looked around the neighbourhood [for vestibular rehabilitation], but no one knows what it is, and, well, they can’t really help me.’* Lastly, long waiting times led to a delay in diagnosis, which prompted the patient to search for other possibilities and could facilitate AVD use.

## Discussion

### Summary

During this qualitative interview study, we comprised barriers and facilitators to appropriate vestibular care in themes that we identified as the competence, mindset, relational determinants, and accessibility to care.

Facilitators to appropriate care included adequate knowledge about vestibular disorders, GPs valuing delivering high-value care, positive experiences with physiotherapy, patients being able to cope with their complaints, personal continuity, close collaboration between GP and colleagues, patients receiving support from their immediate environment and/or fellow patients, and sufficient time and availability of healthcare providers.

Barriers to appropriate care included insecurities about the diagnosis and treatment by GPs, patients doubting the GPs’ competence, patients feeling desperate to try every treatment and GPs wanting to accommodate those wishes, (hearing) positive experiences with AVDs, AVD prescriptions by other healthcare providers, and insurance not covering physiotherapy.

### Strengths and limitations

Strengths of our study included combining perspectives from GPs and patients. Interviews from both groups were conducted concurrently. With each new interview, the interviewer incorporated her findings from the previous interview(s). In doing so, we were able to obtain a comprehensive view on the management of vestibular symptoms. As a qualitative interview study, our goal was to reach a rich understanding rather than statistical representativeness. The sample size of 11 GPs and 15 patients allowed us to achieve thematic completeness for our research aim, consistent with qualitative methodological standards [27]. Additionally, the participants varied in characteristics, which contributed to the comprehensiveness of the data. Furthermore, we supported the objectivity and reliability of our data by having two independent coders and by regularly discussing the results with the whole research team. Lastly, we used the Theoretical Domains Framework [29], a framework to understand behaviour change, to develop the topic list for the interviews. This gave our study a strong theoretical basis and helped us provide a comprehensive coverage of possible barriers and facilitators.

There are also some limitations to acknowledge. Except for one patient, all included patients were recruited through the patient association and they had been dealing with vestibular symptoms for a long time (ranging from a year to decades). Their perspectives could therefore have reflected those of only a specific group. However, some of our findings are comparable to those from other interview studies with patients with vestibular symptoms. This suggests that our participants’ perspectives are in line with those found in the broader population. Another Dutch interview study from 2016 evaluated experiences from patients, aged 65 years and older, with vestibular symptoms [30]. Participants were not recruited through the patient association and did not necessarily have chronic symptoms. Themes that also arose in our study included the impact of vestibular symptoms on daily life (e.g. a reduced mobility), acceptance of the complaints as a coping strategy, and patients doubting the GP’s capabilities. During a German study from 2010, patients aged 65 years and older were interviewed, regardless of the duration of their vestibular symptoms [31]. Similar themes included negative effects of the symptoms (e.g. being unable to receive care due to a reduced mobility), positive effects from receiving support from their social network/fellow patients, and acceptance as a way of coping. Still, it is important to keep in mind that our participants had perhaps a more negative or critical view on the management of their symptoms. A future study may provide new insights by recruiting participants through other sources (e.g. patients treated in secondary care) and/or using focus groups to validate results from interview studies, prioritise issues, and discuss solutions for these issues. Another limitation might be that we did not use ‘experience with (advising or doing) vestibular exercises’ as a criterium during purposive sampling, potentially yielding data less equipped to answer our research question. However, our participating GPs and patients still varied regarding this characteristic: we included both GPs and patients with and without experience.

### Comparison with existing literature

Several qualitative studies regarding either GPs’ or patients’ experiences with (the management of) vestibular symptoms have been conducted. None of those studies combined perspectives from both groups or focused on barriers and facilitators to AVD use/prescriptions and advising/doing vestibular exercises.

Two interview studies (one American study from 2009 and one German study from 2018) described challenges that GPs, and other clinicians, experienced when managing patients with vestibular symptoms [28,32]. Lack of education, knowledge, skills, experience and/or exposure were barriers that GPs and other clinicians experienced. Other factors that were mentioned were: delays in getting a specialist appointment and patients being keen on non-evidence-based medication. On the other hand, exchange with colleagues was mentioned as a facilitator for appropriate care. All aforementioned factors are similar to our findings.

Three interview studies described (older) patients’ experiences with vestibular symptoms and the management of these symptoms in general practice: one German study from 2010, one Swedish study from 2014, and one Dutch study from 2016 [30,31,33]. As mentioned before, many of the themes resemble those that we reported. Important themes were the impact of the symptoms on daily life and acceptance as a coping strategy, as well as trying out different strategies to cure, improve or control the symptoms. Patients also mentioned that receiving support from their social network, their healthcare provider, and/or fellow patients, enhanced physical and psychological aspects. Notably, the studies reported that patients doubted the GPs’ competence, a theme that also recurred in our study.

Lastly, a study reporting a synthesis of qualitative evidence regarding factors influencing de-implementation or continuation of low-value care (e.g. overprescription of antibiotics) showed factors similar to ours [34]. Important factors in that study were the knowledge of patient and provider, the attitude of patient and provider, and patient-provider communication. Comparable to our findings, the desire to meet expectations of the patients played a major role for the provider. Additionally, clinicians were influenced by their colleagues and (lack of) time, while patients were influenced by their social network, and a desire for diagnostic certainty and perceived control.

### Implications for research and/or practice

Our study had several important findings, which can be used as targets for behaviour change. First, both GPs and patients had doubts about the GPs’ competence regarding the diagnosis and management of vestibular symptoms. Increasing knowledge and skills in GPs is therefore essential. We observed that GPs, but also patients, were insufficiently aware that AVDs are not recommended as a general treatment option for vestibular symptoms. At the same time, awareness of VR as an effective and evidence-based alternative, particularly when tailored to a specific vestibular diagnosis, was limited. Increasing familiarity with VR and resolving logistical barriers may help address other issues identified in our study, such as patients feeling desperate to improve their symptoms and GPs wanting to accommodate their wishes. By swiftly providing an effective treatment, we expect that patients will not feel the necessity to try every alternative option (including AVDs). This also addresses our finding that patients value a clear treatment strategy. Furthermore, logistic and financial barriers can be addressed by providing internet-based VR. This can encourage usage as a part of the patients are able to do the exercises themselves at home for free. Randomised controlled trials have shown that internet-based VR is safe and effective [35,36]. We are currently carrying out a nationwide implementation of internet-based VR (Vertigo Training) [37]. We will use the findings of this study to tailor our implementation strategy and increase the effectiveness. During the evaluation, we will investigate the effect of these interventions, which will hopefully help guide other implementation projects.

## Conclusions

During this interview study, we identified barriers and facilitators to appropriate vestibular care for GPs and patients. Several findings can be used as targets for improvement. We found that both GPs and patients had doubts about the GPs’ competence regarding the management of vestibular symptoms. In addition, patients felt desperate to try every treatment to improve their symptoms and GPs wanted to accommodate those wishes. Aforementioned findings may be addressed by increasing knowledge in GPs about VR, an evidence-based treatment for various vestibular disorders, as GPs will be able to provide an effective treatment. We also found that logistic and financial barriers influenced appropriate vestibular care. Internet-based VR may overcome these barriers, as patients are able to do vestibular exercises themselves at home for free.

## Supplementary Material

Supplemental Material

## Data Availability

The datasets generated and/or analysed during the current study are not publicly available due to privacy and ethics restrictions.
