# Supplementary material for: Factors influencing appropriate vestibular care: An interview study with general practitioners and patients
Source: Eur J Gen Pract. 2025 Dec 16;31(1):2600144. doi: 10.1080/13814788.2025.2600144 (PMC12710262; doi:10.1080/13814788.2025.2600144)
Supplement: Supplemental Material [file IGEN_A_2600144_SM5512.zip › suppl_data/ejgp-2025-0185-File005.docx]

**Supplementary tables and figures**

**Box 1.** Definition of bodily distress disorder according to ICD-11 (Code 6C20) <https://icd.who.int/browse/2025-01/mms/en#767044268>

**Diagnostic Requirements**

- The presence of bodily symptoms that are distressing to the individual. Typically, this involves multiple bodily symptoms that may vary over time. Occasionally the focus is limited to a single symptom, usually pain or fatigue.
- Excessive attention is directed toward the symptoms, which may manifest in:
- Persistent preoccupation with the severity of the symptoms or their negative consequences. In individuals who have an established medical condition that may be causing or contributing to the symptoms, the degree of attention related to the symptoms is clearly excessive in relation to the nature and severity of the medical condition.
- Repeated contacts with health care providers related to the bodily symptoms that are substantially in excess of what would be considered medically necessary.
- Excessive attention to the bodily symptoms persists despite appropriate clinical examination and investigations or appropriate reassurance by health care providers.
- Bodily symptoms are persistent; that is, some symptoms are present (though not necessarily the same symptoms) on most days during a period of at least several months (e.g., 3 months or more).
- The bodily symptoms and related distress and preoccupation result in significant impairment in personal, family, social, educational, occupational or other important areas of functioning.
- The symptoms or the associated distress and preoccupation are not better accounted for by another mental disorder (e.g., Schizophrenia or Other Primary Psychotic Disorder, a Mood Disorder, or an Anxiety or Fear-Related Disorder).

**Table 1.** Qualitative analysis of the free-text answers. Each tag (second column) from student quote (first column) was reported separated by a comma.

| **1. Which aspects of your participation in the project do you consider most relevant to improving your well-being?** | |
| --- | --- |
| **Patient’s quote** | **Keywords** |
| More sport and sleep. That I could talk about it and was well looked after. | Physical Exercise, Sleep, Counselling, Assistance |
| Exercise, sleep (I still need sleeping pills) | Movement, Sleep, Drug Therapy |
| Great support at various levels | Support |
| Sport, conscious breaks | Physical Exercise, Rest, Mindfulness |
| Motivation to do sport | Motivation, Physical Exercise |
| 1. change of perspective: sport is a pleasure and not a duty -> over time (relatively early), a healthy need. 2. perception of my body, e.g. heart can beat faster during sport without dying. 3. motivation through very good and personal advice, e.g. alternative exercises. 4. Social contact | Change, Physical Exercise, Joy, No Obligation / Pressure, Healthy Needs, Body Awareness, Motivation, Individual, Advice, Skills, Social Contacts |
| Coaching movement | Coaching, Movement |
| To re-establish a greater awareness of exercise in everyday life. The therapists were always very friendly and helpful. | Awareness, Movement, Routine, Assistance, Therapy |
| Addressing and addressing the problem, increasing sporting activity, eliminating causes | Solutions, Physical Exercise, Eliminate the Causes |
| The hope, the pace and intensity (slow and easy), support of personalized physiotherapies | Hope, Pace, Intensity, No Obligation / Pressure, Individual, Support |
| Awareness of the importance of exercise | Awareness, Movement |
| The importance of sleep and exercise. The analysis and solutions have booked me a lot. | Sleep, Movement, Analysis, Solutions |
| The possibility that I was allowed to participate | Possibility, Participation |
| None. I have taken out a fitness subscription again myself and work out | Physical Exercise, Routine, Self-efficacy, Skills |
| Light fitness training | Physical Exercise, Skills, No Obligation / Pressure |
| Doing more sport in general -> was not directly correlated with the program. Knowing how certain exercises help. | Physical Exercise, Understanding, Skills |
| The security of always having a competent contact person. Objective information about body stress. | Safety, Competence, Counselling, Education, Body Awareness |
| To be generally informed about it | Information |
| Commitment for a self-care moment, physical exercise leading to a more balance of hormones | Commitment, Self-efficacy, Physical Exercise, Balance |
| The company, the discussions with the various experts and the very pleasant atmosphere in the training room | Assistance, Counselling, Competence, Atmosphere, Skills, Room |
| Fitness | Physical Exercise |
| Diagnosis, being taken seriously, movement coach. Exercise / training supervised | Early Detection, Movement, Coaching, Skills, Assistance |
| Reflection - Repetition | Thinking, Routine |
| Approaching body stress as a conscious phenomenon and a holistic method | Stress Reduction, Skills, Body Awareness |
| Immediate onset Fear of sport overcome, relief of symptoms, bridging until psychotherapy | Overcoming Barriers, Physical Exercise, Psychotherapy |
| Psychotherapy helped a lot (talking to professionals about it), group therapy was good to exchange ideas with others | Psychotherapy, Counselling, Group Therapy, Sharing with Others |
| Initially the new sports routine through regular appointments, greater focus on mindfulness | Physical Exercise, Routine, Consultations, Mindfulness |
| Psychiatric care: ADD diagnosed Ritalin helps me a lot | Psychiatry, Assistance, Drug Therapy |
| The understanding of doctors/therapists; sick leave to rest; support through physiotherapy; education | Understanding, Consultations, Rest, Support, Physiotherapy, Education |
| Regular medical consultations Consideration of all my symptoms | Routine, Consultations, Understanding |
| Regular, mandatory appointments, peace and quiet, no pressure, "powering out" | Routine, Discipline, Consultations, Rest, No Obligation / Pressure, Skills |
| Close support for acute stress symptoms. Theoretical input on 'stress' from a psychologist | Assistance, Stress Reduction, Psychology |
| Sport, nutrition, relaxation exercises, sufficient exercise | Physical Exercise, Nutrition, Stress Reduction, Skills |
| Routine sport | Routine, Physical Exercise |
| Movement therapy with physiotherapy. You could feel the change very quickly | Movement, Physiotherapy |
| The feeling of physical pain and of "being sick" (chronic respiratory diseases with and without infection) has become less of a factor in everyday life! The focus has shifted more towards: when and how can I get enough exercise instead of I can't do anything because I feel ill! | Body Awareness, Routine, Mindfulness, Movement |
| Enjoyment of sport is more positive, more energy after sport. Stress reduction feeling after working out. How to practise certain sports positions correctly for the back, stomach,... | Joy, Physical Exercise, Energy, Stress Reduction, Skills |
| physical activity - if possible... | Physical Exercise |
| Sports coaching and motivation with Ms. XX | Physical Exercise, Coaching, Motivation |
| Assessment with questionnaire at the beginning of the consultation Dr. | Assessment, Consultations |
| A real doctor for listening | Counselling |
| Understanding (coaching) helps a lot to take away the fear, regular exercise (slow walking for me), seeing that others feel the same way | Understanding, Coaching, Overcoming Barriers, Routine, Movement, No Obligation / Pressure |
| Understanding the impact of stress, stress reduction | Understanding, Stress Reduction |
| customized plan | Individual, Coaching |
| Being accompanied throughout the project: Helped to regain confidence in my body | Assistance, Body Awareness |
| Regular workouts (strength training), mindfulness yoga, psychiatrist (a few sessions) | Routine, Skills, Mindfulness, Psychiatry, Consultations |
| Self-regulation, relaxation | Self-efficacy, Stress Reduction |
| As I do a lot of sport in my private life, I decided after the first session to try a different therapy | Physical Exercise, Self-efficacy, Therapy |
| Confident conversation partners, physical activity | Counselling, Skills |

| **2.** **Were there any decisive moments in the course of your treatment? If so, can you describe them?** | |
| --- | --- |
| **Patient’s quote** | **Keywords** |
| I realized that I need to pay attention to my limits and relax. I also really felt the positive effects of sport. | Limits, Stress Reduction, Self-efficacy, Physical Exercise |
| When I was able to exercise without any problems, when I adapted my diet | Movement, Nutrition, Self-efficacy |
| Realization that it is best to help yourself | Understanding, Self-efficacy |
| Experience that sport, especially swimming, is good for you. I have corrected my demands on myself. | Experience, Physical Exercise, Self-efficacy |
| 1. discovering rowing (for me like motivation) 2. the feeling of being stronger than I was able to increase the history during training. | Experience, Self-efficacy, Mindfulness, Physical Exercise |
| Realization that even small activities such as walks make a big difference + Kidney stone disease (was a stress trigger) | Self-efficacy, Movement, Stress Factors |
| I found the therapy sessions helpful | Therapy, Counselling, Support |
| Cause: Overload due to work/family, solution; quit job and reorganize | Stress Factors, Solutions, Job, Family, Change |
| Patience, tolerance, acceptance | Endurance, Tolerance, Acceptance |
| Psy. Therapy / Medication | Psychotherapy, Drug Therapy |
| Meeting with doctor and my blockages with exercise and sport. | Consultations, Barriers |
| Communicated verbally: felt seen and viewed holistically | Counselling, Mindfulness, Skills |
| Distance from negative events, osteopathy and cranio / psychotherapy / strength training | Limits, Psychotherapy, Skills, Physical Exercise |
| Stay at the KIZ - calming of the situation. Since then: the symptoms become less "threatening" the better you get to know them, no longer completely out of control. | Understanding, Self-efficacy |
| Motivation | Motivation |
| The most important thing for me is the realization that I have to look for something regular in a group because that is the only thing that helps best. | Understanding, Routine, Counselling |
| When I suddenly did not feel exhausted tired anymore and actually more energized. | Self-efficacy, Energy |
| When I was able to develop a routine and feel the benefits of physical activity | Routine, Body Awareness, Self-efficacy |
| It took a lot of effort to do physiotherapy, but afterwards I felt much better emotionally and much less stressed | Physiotherapy, Barriers, Stress Reduction |
| Realizing + understanding what + why is happening to my body + psyche has been very helpful | Understanding, Body Awareness, Mind, Self-efficacy |
| No individual moments, but a constant confrontation and weighing up of the topic | Routine, Engagement, Thinking |
| Understanding what stress is and what causes it + re-organization. Learning breathing techniques | Understanding, Stress Factors, Change, Breathing Exercises |
| Change of job has significantly reduced stress, realization in psychotherapy less pretending, more being yourself | Job, Change, Stress Factors, Stress Reduction, Understanding, Psychotherapy, Mindfulness |
| After about 3 weeks I noticed a significant improvement in my mental health, which motivated me a lot | Mind, Motivation |
| Ritalin! => more energy, new life head completely different | Drug Therapy, Energy, Change |
| The first visit to Dr. XX: knowing that you are being helped and that you are not alone | Consultations, Assistance |
| The start of my therapy with a psychiatrist | Psychiatry |
| Follow-up appointment every 6 weeks (personal interview, new exercises) | Routine, Counselling, Skills |
| First sporting successes | Physical Exercise, Skills |
| No, I have already carried out the measures before | Skills |
| Birth child | Family |
| 2 weeks after the start of exercise therapy. Improved general condition | Movement, Therapy |
| Yes, physical exertion, getting out of breath no longer feel 'dangerous'! (Trauma from severe scalding as a child and the feeling of not being able to breathe). | Breathing Exercises, Skills, Barriers |
| I lost 3 kg in the first 2 weeks and felt more energetic. Not as much back pain as before. | Energy, Body Awareness |
| Wonderful response from the attending physician. I felt that I was taken seriously and in very good hands. Sports program --> the most important point for me. Coaching by a psychologist | Consultations, Assistance, Physical Exercise, Routine, Counselling, Psychology |
| Cortisol reduction through endurance sports | Stress Reduction, Routine, Physical Exercise |
| Take a break; sleep better, walk better | Rest |
| Muscle building conducive to well-being | Skills, Body Awareness |
| The start of supervised training | Therapy, Movement |
| Learning new habits, breathing exercises, visit to the psychiatrist | Change, Breathing Exercises, Understanding, Psychiatry |
| Different diseases within a short period of time | Comorbidities |
| I realized how good the physical exercise was for me. That it could also distract and activate me to some extent | Body Awareness, Movement, Energy |

| **3. Are there any aspects of treatment that you find critical? If so, which ones?** | |
| --- | --- |
| Patient’s quote | Keywords |
| I have changed my diet myself -> maybe it makes sense to look at this | Nutrition, Education |
| Help from psychologists/psychiatrists must be organized by yourself | Support, Psychology, Psychiatry |
| Routine takes time and patience - perhaps it also takes a little more time. | Time, Routine |
| Little structure, obligation and control | Organization, Discipline, Assistance |
| The information event; those affected could benefit more from better understanding their own situation with stress. No generalizations | Information, Understanding, Stress Factors |
| Motivation to go to training | Motivation |
| It could involve more conversation/ or body therapy | Counselling, Body Awareness |
| Start very slow -> often drive here for only 20min training..., times of physiotherapy very limited, would only recommend it if the person has little idea of sports. | Time, Physiotherapy |
| Personally, physiotherapy didn't help me much, rather repetitive education about symptoms | Physiotherapy, Education |
| It would make sense for me if the movement units were mandatory in movement therapy. | Skills, Discipline |
| The psychological side is not given enough attention. You can certainly achieve a lot through exercise, but I don't think the message that you can solve a lot through exercise (which you hear more and more often) is entirely accurate | Psychology, Skills, Communication |
| Pick up the patient where he/she stands, somatic clarification for the cause | Education |
| More time for patients | Time |
| Relatively little conversation but that's ok, in the end it's physiotherapy | Counselling |
| Body and mind are not really appreciated as one unit | Body Awareness, Psychology, Skills |
| The organization: I thought there was a program but I was supposed to organize everything myself | Organization, Discipline, Assistance |
| It would have been good for me to start psychotherapeutic treatment straight away. It would also be nice to have more support in my search. | Psychotherapy, Support |
| The great difficulty in finding a psychotherapist / psychiatrist. The accusation of not doing enough | Psychology, Psychiatry, Sense of guilt |
| The way performance was measured at the beginning and at the end created unnecessary pressure. | Organization, Communication |
| I can imagine that some patients need even more support | Support |
| I would have liked to have had more discussions with the leading psychiatrist during this time! I felt that one group discussion was not enough! It was very good and very exciting, but you could only scratch the surface a little. | Counselling, Psychiatry |
| Making sport a priority with work. It was difficult for me to stay motivated (work-related). But no criticism of the project. | Routine |
| Possibly more psychological coaching, 1 hour of coaching is/was too little | Psychology |
| Financial or human support | Funding, Support |
| I have benefited most (for years) from Qi Gong / Thai Chi as relaxation and activation techniques. I would include Qi Gong (not just physiotherapy) | Skills, Education |
| Already did a lot of sport / exercise before so no big change | Routine |
| little company but very quickly I stopped feeling ill. No time for everything with work | Assistance, Time |

| **4. Do you believe that the treatment plan will enable you to cope better with physical stress symptoms in the future?   If yes: What specifically will help you?** | |
| --- | --- |
| Patient’s quote | Keywords |
| I know I have support and need to get it. I also want to keep exercising/looking out for symptoms! | Physical Exercise, Early Detection, Self-efficacy |
| Awareness of stress symptoms, the importance of moderate exercise and not overdoing it | Movement, Early Detection |
| Attention to my body | Body Awareness |
| that I have xxx to help myself | Self-efficacy |
| Recognize symptoms, patterns, know possible courses of action | Early Detection, Self-efficacy |
| Exercise regularly | Routine |
| Early detection and direct action | Self-efficacy |
| Interaction between stress and the body better understood, I would take early warning signs more seriously in future. | Early Detection |
| Sleep and exercise as "miracle cures". | Sleep, Movement |
| The interplay of body fitness + mind, self-discipline | Understanding, Mind, Physical Exercise, Self-efficacy, Skills |
| I myself | Self-efficacy |
| Use resources better ¬ faster | Self-efficacy, Early Detection |
| Regular sport | Routine |
| Early recognition of symptoms (for me: digestion); knowing that I react to stress in this way -> more caution | Understanding, Early Detection |
| Activities | Skills |
| Recognize the symptoms & know how to counteract them | Self-efficacy, Understanding, Skills |
| The realization that I need regular classes. Moving, even on a small scale. | Understanding, Routine, Movement |
| Exercise more | Physical Exercise |
| I can categorize my symptoms better and have a few methods to deal with them better | Understanding, Self-efficacy, Skills |
| Mindfulness, regular exercise / training | Mindfulness, Routine, Movement |
| Sport | Physical Exercise |
| Massage, Sport | Massage, Physical Exercise |
| Listen better to signals from the body | Early Detection |
| Breathing technique, relaxation exercise | Mindfulness, Breathing Exercises |
| Better knowledge of what triggers them, active avoidance or better dosage of triggering | Self-efficacy, Understanding, Skills |
| I now know methods that make it easier for me not to get too stressed in stressful situations | Skills, Self-efficacy |
| Ritalin + sport + psychological issues | Drug Therapy, Physical Exercise, Psychology |
| Recognize warning signs earlier and listen to your body, balance stress with exercise | Early Detection, Body Awareness, Physical Exercise, Stress Reduction |
| Perceive signals more quickly, got to know new exercises/sports, recognized the importance of "exercise/time for myself" | Early Detection, Skills, Physical Exercise, Movement |
| to know how much sport and exercise improve general well-being | Understanding, Physical Exercise, Movement |
| The self-awareness and above all the information received | Understanding, Self-efficacy |
| Confirmation that I'm already on the right track anyway | Support |
| Routines | Routine |
| Look and reflect better and intensify physical activities | Thinking, Skills |
| Sport (and therefore feeling my body more) doesn't scare me as much as it gives me the feeling that I have a tool in my own hands to feel better! Self-efficacy | Physical Exercise, Body Awareness, Skills, Self-efficacy |
| Breathing exercises help me to calm down a lot and light exercises. I always feel more positive afterwards. | Breathing Exercises, Skills |
| Regular sport, mental boundaries, being able to say NO sometimes | Routine, Physical Exercise, Limits |
| Recognizing early warning signs, prevention | Early Detection, Prevention |
| I decided to have physiotherapy, which helped me a lot | Physiotherapy |
| This project alone rather not, only including intensive psychotherapy | Psychotherapy |
| Incorporate regular exercise into everyday life | Routine, Movement |
| The realization that it is very helpful to keep moving and exercising | Understanding |
| Breathe properly, mindfulness, positive attitude, more understanding | Breathing Exercises, Mindfulness, Skills, Understanding |
| Better classify the causes of discomfort | Understanding |
| Breathing and physical exercises | Breathing Exercises, Skills, Movement |

**
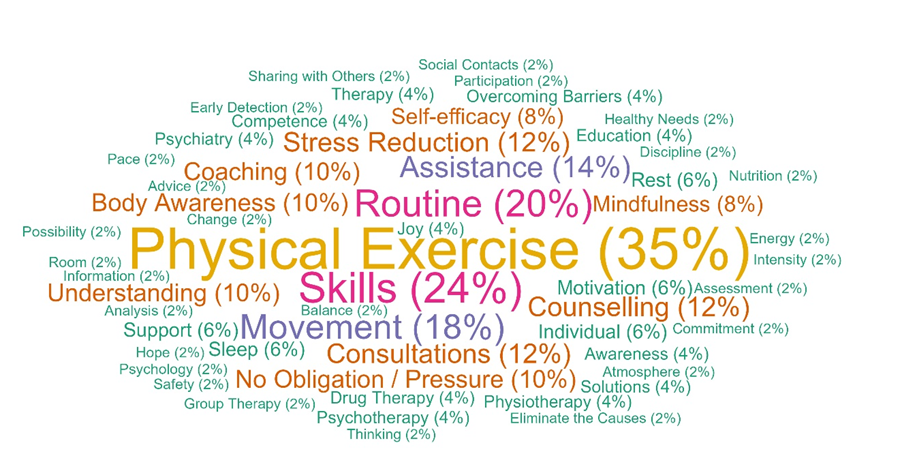
Figure 1**. Tag cloud of factors relevant to improving wellbeing in patients with bodily distress disorder.

Notes. Tags were assigned from responses to the survey question "Which aspects of your participation in the project do you consider most relevant to improving your well-being?”All tags were defined in Supplementary Material 1, Table 1. The percentages were based on the number of patients who answered the questions, N=51, and may not add up to 100%.

**Figure 2**. Tag cloud of key moments/factors in patients' treatment for bodily distress.


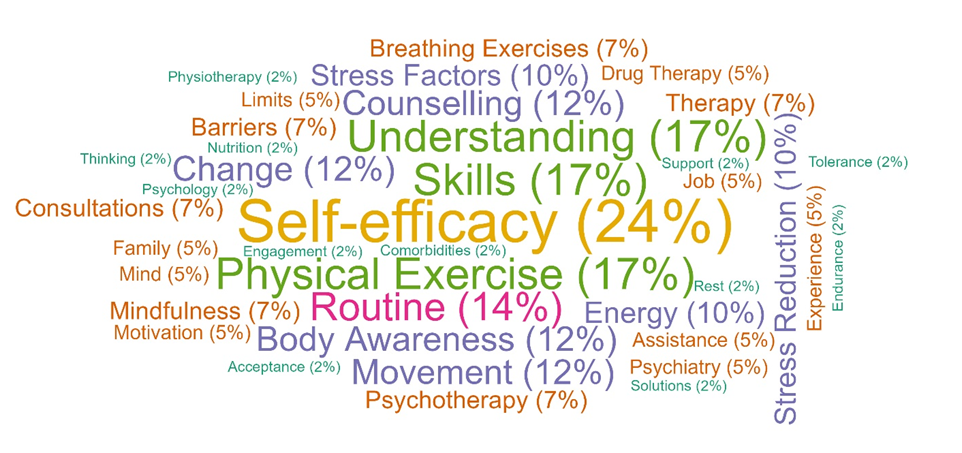


Notes. Tags were assigned from responses to the survey question "Were there any decisive moments in the course of your treatment? If so, can you describe them?” All tags were defined in Supplementary Table 1. The percentages were based on the number of patients who answered the questions, N=42, and may not add up to 100%.

**Figure 3.** Tag cloud of what could be better / what to focus on in future interventions for bodily distress disorder.


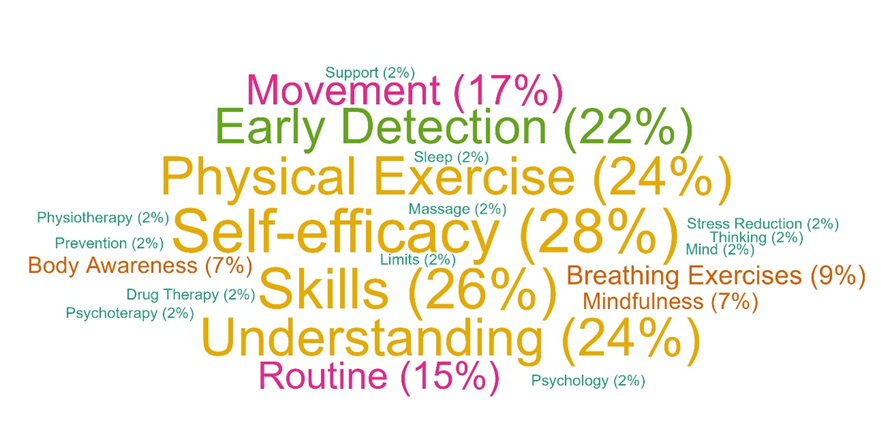


Notes. Tags were assigned based on responses to the survey question "As a result of the treatment program, what specifically will help you better manage physical stress symptoms in the future?" All tags were defined in Supplementary Table 1. The percentages were based on the number of patients who answered the questions, N=46, and may not add up to 100%.

**Figure 4**. Tag cloud of aspects of treatment for bodily distress disorder that patients considered critical, important or in need of more support.


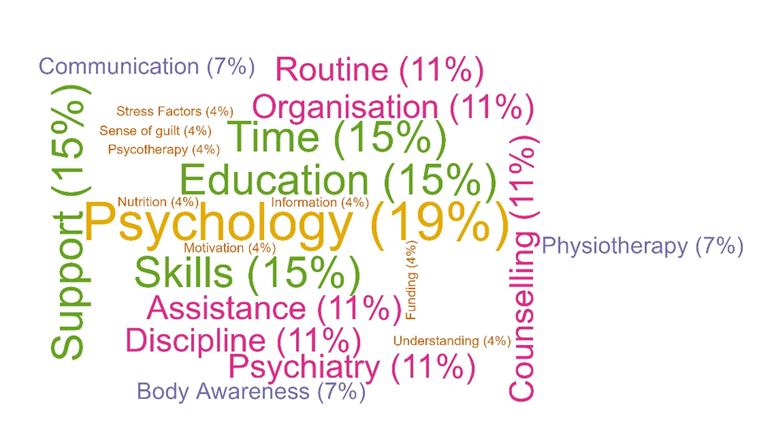


Notes. Tags were assigned from responses to the survey question "Are there any aspects of treatment that you find critical? If so, which ones?" All tags were defined in Supplementary Table 1. The percentages were based on the number of patients who answered the questions, N=27, and may not add up to 100%.

**Table 2**. General practitioners pre (baseline) and post (follow-up) survey. The number of non-missing observations, N, was reported where necessary.

|  |  | Baseline (pre) | Follow-up (post) |
| --- | --- | --- | --- |
| n |  | 11 | 9 |
| Age (mean (SD)) |  | 49.73 (9.46) (min=36, max=67) | 46.33 (8.49)  (min=36, max=61) |
| Sex n(%) | Female | 6 (54.5) | 4 (44.4) |
|  | Male | 5 (45.5) | 5 (55.6) |
| Experience as a family doctor (mean (SD)) | | 10.27 (6.99)  (min=2, max=24) | 10.56 (6.37)  (min=3, max=20) |
| Workload (median (IQR)) | | 60 [60, 75]  (min=40, max=80) | 60 [60, 60]  (min=50, max=80) |
| How interested are you in psychosocial aspects of family medicine? n(%) | Medium | 3 (27.3) |  |
|  | Strong | 7 (63.6) |  |
|  | Little | 1 (9.1) |  |
| On a scale from 0 (very uncertain) to 100 (very certain), how confident do you feel in diagnosing patients in whom you suspect a "psycho-somatic" cause in the sense of a stress disorder? (median [IQR]) | | 70 [50, 73]  (min=10, max=80) | 85 [81, 90]*  (min=60, max=90) |
| What makes the diagnosis of "psycho-somatic" disorder, in the sense of a bodily distress disorder, difficult? – multiple choice-n(%) | | | |
| There are no clear diagnostic guidelines. | | 5 (45.5) |  |
| This is a diagnosis of exclusion. | | 9 (81.8) |  |
| The diagnosis is incomprehensible to patients. | | 6 (54.5) |  |
| The rather unspecific symptoms are difficult to detect. | | 6 (54.5) |  |
| Other reasons. | | - |  |
| To what extent are the following reasons why you did not turn the conversation to a possible bodily distress disorder? (0 = does not apply at all; 10 = fully applies) (median [IQR]) | | | |
| I still don't feel professionally competent in the field. | | | 1 [0, 3]  (min=0, max=9) N=8 |
| I don't manage to bridge the gap in the conversation and address the issue. | | | 2 [1, 5]  (min=0, max=7) N=8 |
| Taking care of bodily distress disorder patients is time-consuming. My consultation hours are simply too busy. | | | 6 [5,8]  (min=1, max=10) N=8 |
| Which elements helped you feel better when dealing with bodily distress disorder patients?  Please choose your top 3 elements n(%) | | | |
| Better knowledge of diagnostics | | | 6 (66.7) |
| More self-confidence in terms of my competence | | | 7 (77.8) |
| That I could offer the patient something, e.g. physiotherapy | | | 5 (55.6) |
| A clear algorithm that I can stick to | | | 5 (55.6) |
| Tips and tricks for conducting conversations | | | - |
| The exchange with colleagues on the topic | | | 1 (11.1) |
| Other | | | - |
| What would help you feel better when dealing with bodily distress disorder patients? Please choose your top 3 elements n(%) | | | |
| Communication trainings | | | 5 (55.6) |
| Specific suggestions | | | 2 (22.2) |
| Simply more routine | | | 4 (44.4) |
| More practical access to the materials | | | 1 (11.1) |
| Regular exchange with colleagues | | | 3 (33.3) |
| Professional supervision and mentoring | | | 1 (11.1) |
| More information material for patients | | | 4 (44.4) |
| A video for patients in which the topic is also explained visually | | | 3 (33.3) |
| Other | | | 1 (11.1) |
| On a scale from 0 (very uncertain) to 100 (very certain), how confident do you feel in treating patients for whom you suspect a "psycho-somatic" cause in the sense of a stress disorder? (median [IQR]) | | 50 [38, 66]  (min=0, max=74) | 82 [66, 85]**  (min=50, max=90) |
| What therapeutic measures do you usually take (i.e. in > 50% of all cases) for symptoms that you consider to be probably psychological or stress-related n(%) | | | |
| I explain the cause of the complaints to the patient in detail | | 8 (72.7) |  |
| I dispense psychotropic drugs | | 2 (18.2) |  |
| I dispense Phyto therapeutics | | 5 (45.5) |  |
| I refer the patient to a family doctor who is interested in psychosomatic medicine | | 2 (18.2) |  |
| I send the patient to a psychiatrist for a consultation | | 1 (9.1) |  |
| I send the patient to a psychiatrist | | 1 (9.1) |  |
| I send the patient to a psychologist | | 7 (63.6) |  |
| I refer the patient to a complementary medicine practitioner | | - |  |
| I refer the patient to a complementary therapist (such as breathing therapy, shiatsu, q-gong, etc.) | | -  3 (27.3) |  |
| I prescribe physiotherapy and/or physical training | | 8 (72.7) |  |
| I treat the accompanying sleep disorder | | 3 (27.3) |  |
| I prescribe meditation or other mindfulness techniques | | - |  |
| I'm doing nothing for now and waiting for a spontaneous recovery in the sense of "watchful waiting" | | 1 (9.1) |  |
| I prescribe painkillers | | - |  |
| Other measures | | - |  |
| When you think about the last 3 months - how good was your experience of working with the physiotherapists?  (0 = very bad; 10 = very good) (median [IQR]) | | 8 [8, 9]  (min=6, max=9) | 8 [8, 9]  (min=4, max=9) |
| How often do you prescribe physiotherapy for "psycho-somatic" patients with bodily distress disorder? n(%) | Never  Rare / occasionally  Regularly / frequently | 2 (18.2)  7 (63.6)  2 (18.2) | N=8  -  3 (37.5)  5 (62.5) |
| If you think back over the last 3 months, how often have you received feedback from the physiotherapists treating patients for whom you prescribed physiotherapy on the progress of their therapy? n(%) | | -  8 (72.7)  3 (27.3) | N=8  2 (25.0)  5 (62.5)  1 (12.5) |
| Never  Rare / occasionally  Regularly / frequently | |  |  |
| In your opinion, is increased cooperation with physiotherapy in the treatment of bodily distress disorder desirable? n%) | No  Yes | 2 (18.2)  9 (81.8) |  |
|  |  |  |  |
| In your opinion, is there a need for cooperation with other professional groups such as body therapists, respiratory therapists, psychologists, etc.? n(%) | No  Yes | 2 (18.2)  9 (81.8) |  |
| I will continue to use the model in my consultation hours insert.  (0 = Does not apply at all; 10 = Fully applies) (median[IQR]) | | | 10 [9, 10]  (min=6, max=10) |
| I would recommend the use of the model to family doctor friends. Recommend to colleagues. (0 = Does not apply at all; 10 = Fully applies) (median[IQR]) | | | 10 [9, 10]  (min=9, max=10) |
| The reactions of my patients with regard to the following. Measures were particularly positive  (multiple answers possible) n(%) | | | |
| The doctor-patient conversation on the topic of bodily distress itself | | | 8 (88.9) |
| Education using the materials | | | 5 (55.6) |
| Group coaching | | | 4 (44.4) |
| Physiotherapy | | | 5 (55.6) |
| The use of PRISM | | | 2 (22.2) |
| Sleep therapy | | | 4 (44.4) |

Abbreviations. SD: standard deviation; IQR: interquartile range; min: minimum; max: maximum.

* significant difference post-pre p=0.006

** significant difference post-pre p=0.005

**Table 3.** Physiotherapists pre (baseline) and post (follow-up) survey. The number of non-missing observations, N, was reported where necessary.

|  |  | Baseline (pre) | Follow-up (post) |
| --- | --- | --- | --- |
| n |  | 6 | 3 |
| Age (mean (SD)) |  | 36.83 (8.52) (min=27, max=46) | 39 (10)  (min=27, max=46) |
| Gender n(%) | female | 6 (100.0) | 3 (100.0) |
| Experience as a physiotherapist (mean (SD)) |  | 8.83 (8.61)  (min=1, max=20) | 11 (8)  (min=4, max=20) |
| Workload (median [IQR]) |  | 72 [61, 88]  (min=60, max=90) | 80 [80, 85]  (min=80, max=90) |
| How interested are you in the psychosocial aspects of physiotherapy? n(%) | medium  strong | 2 ( 33.3)  4 ( 66.7) |  |
| On a scale from 0 (very uncertain) to 100 (very certain), how confident do you feel about assessing patients for whom you suspect a "psycho-somatic" co-cause? (median [IQR]) |  | 66 [57, 70]  (min=15, max=80) |  |
| On a scale from 0 (very uncertain) to 100 (very certain), how confident do you feel in treating patients for whom you also suspect a "psycho-somatic" cause of the complaints? (median [IQR]) |  | 65 [45, 72] (min=15, max=74) |  |
| I now feel more confident in dealing with patients with body stress symptoms (0 = does not apply at all; 10 = fully applies) (median [IQR]) |  |  | 9 (9,9)  (min=9, max=9) |
| How strong is your motivation to get involved in the treatment "psycho-somatic" patients? (0 = very low; 100= very high) (median [IQR]) |  | 85 [73, 90] (min=69, max=92) |  |
| Do you need training in dealing with "psycho-somatic" patients? n(%) | No  Yes | 1 (16.7)  5 (83.3) |  |
| I have taken part in training courses on the subject of bodily distress disorder n(%) | | No  Yes | -  3 (100) |
| Is the information provided by the referring physician (prescriptions) sufficient for good treatment planning for body stress disorder? (0 = does not apply at all; 10 = fully applies) (median [IQR]) |  | 50 [50, 50]  (min=38, max=55) |  |
| In your opinion, would you like to see more cooperation between doctors and physiotherapy for the treatment of body stress disorder? n(%) | No  Yes | 1 ( 16.7)  5 ( 83.3) |  |
| To what extent has cooperation with the GPs changed as a result of the project? n(%) | Nothing has changed  There is more exchange than before | | 1 ( 33.3)  2 (66.7) |
| Thinking about the last 3 months, how good was your experience of working with the doctors in treating "psycho-somatic" patients? (0 = very bad; 100 = very good) (median [IQR]) (median (SD)) |  | 50 [42, 50]  (min=39, max=63) |  |
| In your opinion, is there a need for better cooperation with "psycho-somatic" patients? (%) | No  Yes | 1 ( 16.7)  5 ( 83.3) |  |

Abbreviations. SD: standard deviation; IQR: interquartile range; min: minimum; max: maximum; GPs: general practitioners.
