# Supplementary material for: Factors influencing appropriate vestibular care: An interview study with general practitioners and patients
Source: Eur J Gen Pract. 2025 Dec 16;31(1):2600144. doi: 10.1080/13814788.2025.2600144 (PMC12710262; doi:10.1080/13814788.2025.2600144)
Supplement: Supplemental Material [file IGEN_A_2600144_SM5512.zip › suppl_data/ejgp-2025-0185-File006.pdf]

1. Example of materials used for patient education

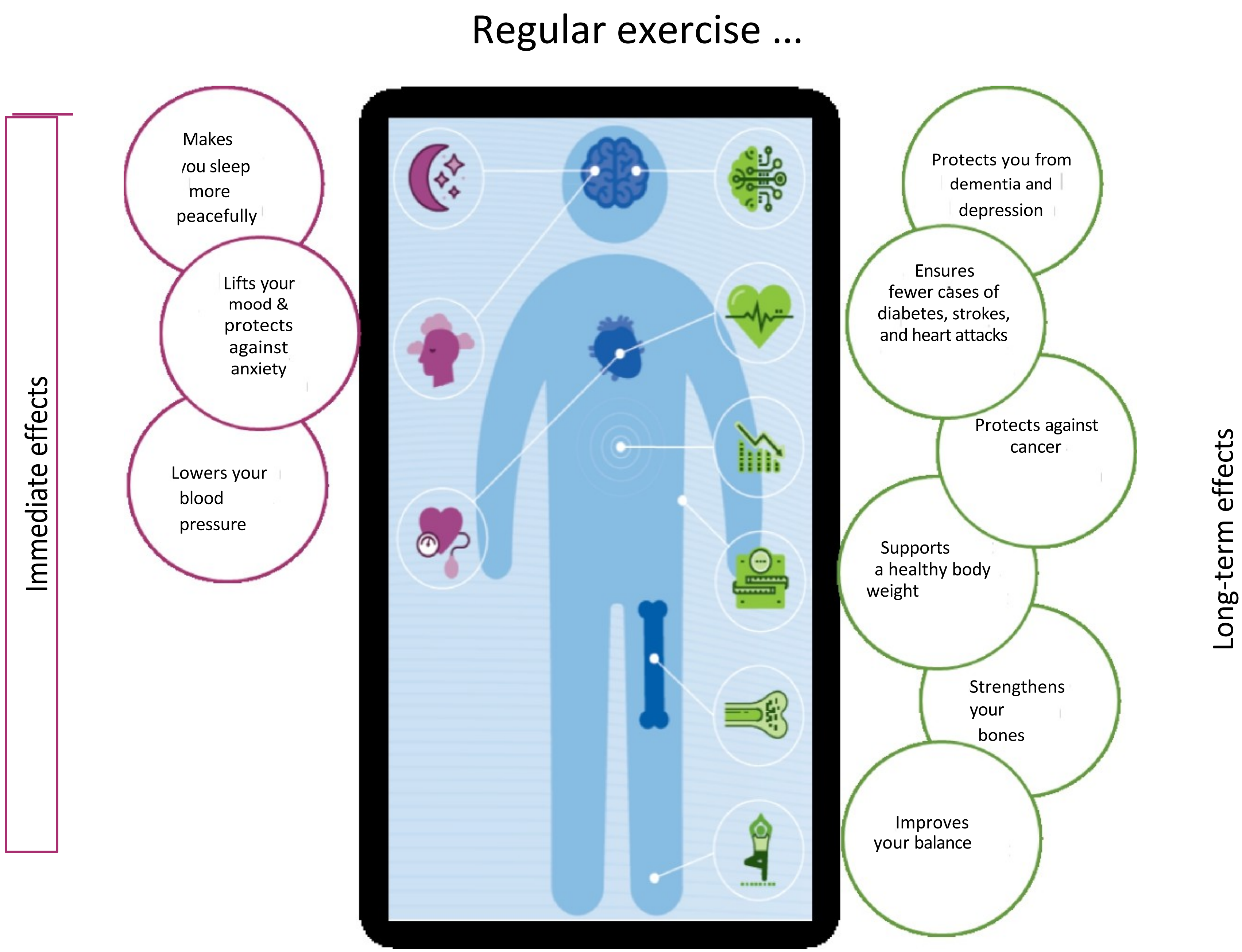

How can stress manifest itself physically?

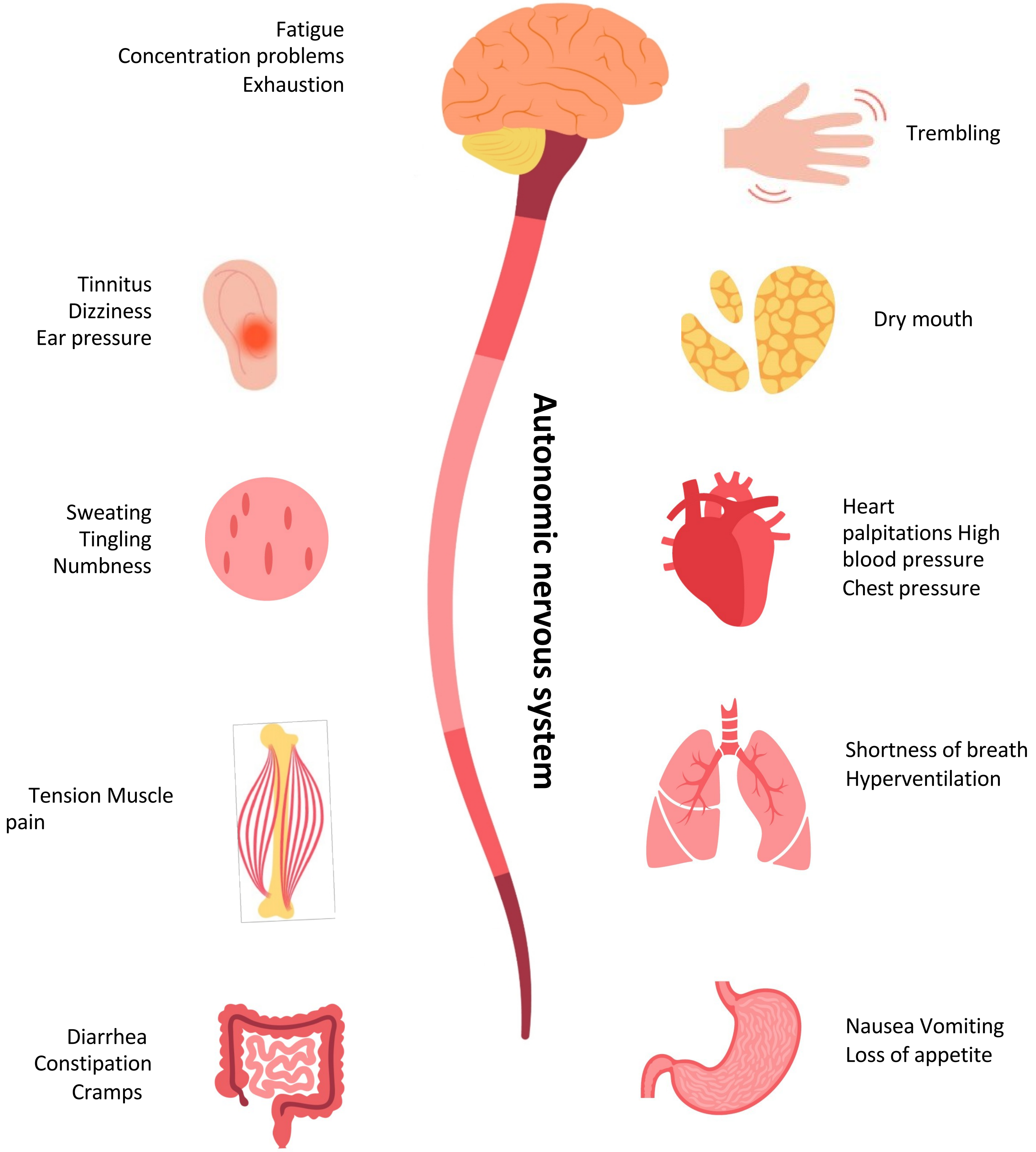

Dear patients,

You have been taking part in our body stress project for around 6 months due to physical and perhaps also mental stress symptoms. We are trying to improve the interdisciplinary treatment of the consequences of stress. Your experiences are essential for the further development of the treatment.

We are interested on the one hand in the development of your complaints and on the other hand in your experiences during the project.

We therefore kindly ask you to answer the following questions and return the questionnaire to us in the way that is most convenient for you:

- Hand in at reception or to the doctor in charge during the next consultation
- Send to the practice by post
- Upload via the contact form.

If you have any questions, please contact us at any time using the contact form.

Your answers will be treated confidentially and published in a summarized form (without drawing conclusions about individual participants). With your participation you agree to this.

We would like to thank you for your contribution and wish you all the best for your health!

First name: _________________ Nachname: _________________________________

Date of birth: _____________ Date: _______________

Questions about your state of health

**PHQ-15: Patient Health Questionnaire 15-item Somatic Symptom Severity Scale**			
To what extent have you felt affected by the following complaints in the last 4 weeks?			
	Not impaired<br>0 points	Little affected<br>1 point	Severely impaired<br>2 points
Abdominal pain	☐	☐	☐
Back pain	☐	☐	☐
Pain in arms, legs or joints (knees, hips, ...)	☐	☐	☐
Menstrual pain or other menstrual problems (women only)	☐	☐	☐
Headache	☐	☐	☐
Pain in the chest area	☐	☐	☐
Dizziness	☐	☐	☐
Fainting spells	☐	☐	☐
Palpitations or tachycardia	☐	☐	☐
Shortness of breath	☐	☐	☐
Pain or problems during sexual intercourse	☐	☐	☐
Constipation, nervous bowel or diarrhea	☐	☐	☐
Nausea, flatulence or indigestion	☐	☐	☐
Tiredness or feeling of having no energy	☐	☐	☐
Difficulty falling asleep or staying asleep, or increased sleep	☐	☐	☐
Total: ____/30 points			

**Physical Activity Vital Sign (PAVS)**	
How many days a week do you carry out physical activity of moderate or high intensity (e.g. fast walking)?	_____ Days/week
How many minutes do you do these physical activities on average?	_____ minutes
Total time of moderate physical activity per week	_____ min / week

Questions about your experience in the project

1. **Which aspects of your participation in the project do you consider particularly relevant to improving your well-being?**

   ____

   ____

   ____

2. **Were there any decisive moments in the course of your treatment? If so, can you describe them?**

   ____

   ____

   ____

3. **Are there any aspects of treatment that you find critical? If so, which ones?**

   ____

   ____

   ____

4. **Could you explain what bodily distress disorder is to someone you know today in an understandable way? Please mark with a cross.**

   Yes ________ No ________

5. **A challenging question: If you had to assign your physical symptoms to different causes, what percentage would you assign to the following aspects (100% in total)?**

   Psyche: ________ %

   Body: ________ %

   Stress ________ %

6. **How has the impairment caused by the bodily distress disorder symptoms changed in the last 6 months? Please mark with a cross.**

-100%	-80%	-60%	-40%	-20%	0<br>Initial situation	+20%	+40%	+60%	+80%	+100%
Decrease in symptoms						Increase in symptoms				

7. **Do you believe that the treatment plan will enable you to cope better with physical stress symptoms in the future? Please mark with a cross.**

Yes ________ No ________

If yes, what specifically will help you?

____

____

8. **The medical consultation with the explanations and illustrative materials was helpful.**

0	1	2	3	4	5	6	7	8	9	10
Absolute at all not applicable										applicable

9. **I found the discussions with the physiotherapist helpful.**

0	1	2	3	4	5	6	7	8	9	10
Absolute at all not applicable										applicable

10. **I found the training with physiotherapy helpful.**

0	1	2	3	4	5	6	7	8	9	10
Absolute at all not applicable										applicable

11. **I noticed that the doctor and physiotherapist worked together as a team to manage my treatment.**

0	1	2	3	4	5	6	7	8	9	10
Absolute at all not applicable										applicable

12. **Would you recommend participation in such a project to friends and acquaintances?**

0	1	2	3	4	5	6	7	8	9	10
Absolute at all not										

3. Survey to general practitioners

Languages	
ID	Display Name
en	German (default)

	C	Variable / Field Name	Field Label<br>***Field Note***	**Field Attributes (Field Type, Validation, Choices, Calculations, etc.)**
**Instrument:** **Pre-treatment survey**<br>Active languages - Data Entry: en| Survey: en				
	1	[record_id]	Record ID	text
	2	[begin]	Please click on the "Next page" button to start filling in the form.	descriptive, Required
	3	[a1]	Section Header:<br>Are you one of the doctors involved in the project?	yesno, Required<br>1 Yes<br>0 No<br>Custom alignment: LV
	4	[a2_1]	Section Heade *t: Socio-demographic data*<br>Your year	text (integer, Min: 1 900, Max: 2022), Required<br>Custom alignment: RH<br>Field Annotation: @PLACEHOLDER='e.g. 1979' AND @CHARLIMIT=4
	5	[a2_2]	Gender	radio, Required<br>1 female<br>2 male<br>3 diverse
	6	[a2_3]	Experience as a general practitioner	text (integer, Min: 1, Max: 99), Required<br>Custom alignment: RH<br>Field annotation: @PLACEHOLDER='years'
	7	[a2_4]	Workload	text (integer, Min: 0, Max: 100), Required<br>Custom alignment: RH<br>Field annotation: @PLACEHOLDER='percent'
	8	[a2_5]	Specialist in internal medicine/family medicine	yesno, Required<br>1 Yes<br>0 No
	9	[a2_6]	Certificate of Proficiency in Psychosomatic and Psychosocial Medicine (FAPPM)	yesno, Required<br>1 Yes<br>0 No
	10	[a7]	Section Header: *Assessments on the diagnosis and treatment of body stress disorder*<br>How interested are you in the psychosocial aspects of family medicine?	radio, Required<br>1 little<br>2 medium<br>3 strong
	1J	[a8]	How many of the patients you have treated in the last week do you suspect have a "psycho-somatic" cause in the sense of a physical stress disorder?	radio, Required<br>1 With none/none<br>2 1 to 5<br>3 6 to J 0<br>4 For more than 10

	12	[a9]	Section Header:<br>On a scale from 0 (very uncertain) to 1 00 (very certain), how confident do you feel in diagnosing patients in whom you suspect a "psycho-somatic" cause in the sense of a stress disorder?	slider (number, Min: 0, Max: 100), Required<br>Slider labels: very uncertain, very certain<br>Custom alignment: RH
	13	[a10]	What makes the diagnosis of "psycho-somatic" Disorders in the sense of a body stress disorder difficult?(multiple answers possible)	checkbox, Required<br>1 | a1 0 1 | There are no clear diagnostic guidelines<br>2 | a1 0 2 | This is a diagnosis of exclusion<br>3 | a10 3 | The diagnosis of "body stress" is incomprehensible to patients<br>4 | a10 4 | The rather unspecific symptoms are difficult to detect<br>5 | a1 0 5 | Other reasons
	14	[a10_text]<br>Show the field ONLY if:<br>[a10(5)] = '1'	Describe the reasons:	notes<br>Custom alignment: LH
	15	[a11]	On a scale from 0 (very uncertain) to 100 (very certain), how confident do you feel in treating patients who you suspect have a "psycho-somatic" cause in the sense of a stress disorder?	slider (number, Min: 0, Max: 1 00),<br>Required Slider labels: very uncertain,<br>very certain Custom alignment: RN
	16	[a12]	Senion Header:<br>What therapeutic measures do you usually take (i.e. in > 5096 of all cases) for symptoms that you to be probably psychological or stress-related? (multiple answers possible)	checkbox, Required<br>1 | a12 1 | I explain the cause of the complaints to the patient in detail<br>2 | a12 2 | I dispense psychotropic drugs<br>3 | a12 3 | I dispense phytotherapeutics<br>4 | a12 4 | I refer the patient to a family doctor who is interested in psychosomatic medicine<br>5 | a12 5 | I send the patient to a psychiatrist for a consultation<br>6 | a12 6 | I send the patient to a psychiatrist<br>7 | a12 7 | I send the patient to a psychologist<br>8 | a12 8 | I refer the patient to a complementary medicine practitioner<br>9 | a12 9 | I refer the patient to a complementary therapist (such as breathing therapy, shiatsu, q-gong, etc.)<br>10 | a12 10 | I prescribe physiotherapy and/or physical training<br>11 | a12 11 | I treat the accompanying sleep disorder<br>12 | a12 12 | I prescribe meditation or other mindfulness techniques<br>13 | a12 13 | I'm doing nothing for now and waiting for a spontaneous recovery in the sense of "watchful waiting"<br>14 | a12 14 | I prescribe painkillers<br>15 | a12 15 | Other measures<br>Custom alignment: LV
	17	[a12_text]<br>Show the field ONLY if:<br>[a12(15)] = '1'	What other measures?	notes, Required<br>Custom alignment: LH
	18	[a13]	Section headset: *interprofessional collaboration with physiotherapy*<br>How many patients have you sent to physiotherapy in the last 3 months?'(approximate estimate)	radio, Required<br>1 | 0 to 5<br>2 | 6 to 1 0<br>3 | 1 1 to 20<br>4 | 21 to 40<br>5 | more than

				40
	19	[a14]	When you think about the last 3 months - how good was your experience of working with the physiotherapists?	slider (number, Min: 0, Max: 1 00), Required<br>Slider labels: very bad, very good Custom alignment: RH
	20	[a15]	If you think back over the last 3 months how often have you received feedback from the physiotherapists treating patients for whom you prescribed physiotherapy on the progress of their therapy?	radio, Required<br>1 never<br>2 rare<br>3 regularly
	21	[a16]	Senion Header:<br>How often do you prescribe physiotherapy for "psychosomatic" patients with body stress disorder?	radio, Required<br>1 never<br>2 rare<br>3 regular
	22	[a17]	To what extent do you agree with the physiotherapists on the diagnosis and treatment of body stress disorder?	radio, Required<br>1 Consensus between doctors and physiotherapists rarely exists<br>2 Consensus usually exists between doctors and physiotherapists<br>3 I do not know
	23	[a18]	In your opinion, is increased cooperation with physiotherapy in the treatment of body stress disorder desirable?	yesno, Required<br>1 Yes<br>0 No
	24	[a18_2]<br>Show the field ONLY if:<br>[a18] = '1'	What measures could be taken to improve cooperation physiotherapists?	notes<br>Custom alignment: LH
	2S	[a19]	In your opinion, is there a need for cooperation with other professional groups such as body therapists, respiratory therapists, psychologists, etc.*?	yesno, Required<br>1 Yes<br>0 No
	26	[a19_2]<br>Show the field ONLY if:<br>[a19] = '1 '	Which other professional groups should be included?	notes<br>Custom alignment: LH
	27	[text2]	Senion Header:<br>Thank you for answering the questions, please click on the "Submit" button complete the questionnaire.	descriptive
	28	[gp_complete]	Section Header: *Farm Status*<br>Complete?	dropdown<br>0 Incomplete<br>1 Unverified<br>2 Complete

Instruments	Languages

	#	Variable / Field Name	Field Label<br>*Field Note*	Field Attributes (Field Type, Validation, Choices, Calculations, etc.)
**Instrument: Post-treatment survey**				**Enabled as survey**
		Active languages - Data Entry: en| Survey: en		
	1	[record_id]	Record ID	text
	2	[begin]	Please click on the "Next page" button to start filling in the form.	descriptive, Required
	3	[pilot]	Section Header:<br>Are you one of the doctors in the project?	yesno, Required<br>1 Yes<br>0 No
	4	[a1]	Section Header: *Socio-demographic information*<br>1) Your year	text (integer, Min: 1900, Max: 2022), Required<br>Custom alignment: RH<br>Field Annotation: @PLACEHOLDER='e.g. 1979' AND @CHARLIMIT=4
	5	[a2]	2) Gender	radio, Required<br>1 female<br>2 male<br>3 diverse
	6	[a3]	3) Experience as a general practitioner	text (integer, Min: 1, Max: 99), Required<br>Custom alignment: RH<br>Field annotation: @PLACEHOLDER='years'
	7	[a4]	4) Workload	text (integer, Min: 0, Max: 100), Required<br>Custom alignment: RH<br>Field annotation: @PLACEHOLDER='percent'
	8	[a5]	Section Header: *Socio-demographic data (continued)*<br>5) Certificate of competence in "Psychosomatic and Psychosocial Medicine" (FAPPM)	yesno, Required<br>1 Yes<br>0 No
	9	[a6]	6) I took part in the training courses and sessions on the subject of "body stress".	yesno, Required<br>1 Yes<br>0 No
	10	[a7]	8) I already took part in the first survey.	yesno, Required<br>1 Yes<br>0 No
	11	[a8]	9) I have been able to include about this many patients in the project:	radio<br>1 none<br>2 1-5<br>3 6-10<br>4 more than 10
	12	[b1]	Section Header: *Assessment of diagnostics and treatment of body stress*<br>10) How many of your patients that you have treated in the last (working) week do you suspect a "psychosomatic" cause in the sense of a physical stress disorder?	text (integer, Min: 0, Max: 100), Required<br>Field annotation: @PLACEHOLDER='Number of patients'
	13	[b2]	11) On a scale from 0 (very uncertain) to 100 (very certain), how confident do you feel in diagnosing patients in whom you suspect a "psycho-somatic" cause in the sense of a body stress disorder?	slider (number, Min: 0, Max: 100), Required<br>Slider labels: 0, , 100<br>Custom alignment: RH
	14	[b3]	12) On a scale from 0 (very uncertain) to 100 (very certain), how confident do you feel in treating patients who you suspect to have a "psycho-somatic" cause in the sense of a body stress disorder?	slider (number, Min: 0, Max: 100), Required<br>Slider labels: 0, , 100<br>Custom alignment: RH
	15	[text_01]	Section Header:<br>13) To what extent are the following reasons why affected patients were not included in the project?	descriptive
	16	[b4]	a) No willingness on the part of the patients0 = Does not at all; 10 = Fully applies	radio, Required<br>0 0<br>1 1<br>2 2<br>3 3<br>4 4

				5 5<br>6 6<br>7 7<br>8 8<br>9 9<br>10 10<br><br>Custom alignment: LH
	17	[b5]	b) I simply did not have time to inform the patient sufficiently0 = Does not apply at all; 10= Fully applies	radio, Required<br>0 0<br>1 1<br>2 2<br>3 3<br>4 4<br>5 5<br>6 6<br>7 7<br>8 8<br>9 9<br>10 10<br><br>Custom alignment: LH
	18	[b6]	14) What is your estimate of the percentage of patients for whom you now suspect a functional problem but have decided not to go into more detail now or in a second consultation?<br><br>Section Header:	text (integer, Min: 0, Max: 100), Required<br>Field annotation: @PLACEHOLDER='in percent'
	19	[text_02]	15) To what extent are the following reasons why you not turn the conversation to a possible physical stress disorder? 0 = Does not apply at all; 10 = Fully applies	descriptive
	20	[b7]	a) I still don't feel professionally competent in the diagnosis and treatment of body stress.	radio, Required<br>0 0<br>1 1<br>2 2<br>3 3<br>4 4<br>5 5<br>6 6<br>7 7<br>8 8<br>9 9<br>10 10<br><br>Custom alignment: LH
	21	[b8]	b) I don't manage to bridge the gap in the conversation and address the issue.	radio, Required<br>0 0<br>1 1<br>2 2<br>3 3<br>4 4<br>5 5<br>6 6<br>7 7<br>8 8<br>9 9<br>10 10<br><br>Custom alignment: LH
	22	[b9]	c) Taking care body stress patients is time-consuming. My consultation hours are simply too busy.	radio, Required<br>0 0<br>1 1<br>2 2<br>3 3<br>4 4

				5 5<br>6 6<br>7 7<br>8 8<br>9 9<br>10 10<br><br>Custom alignment: LH
23	[b10]		Section Header:<br>16) Which elements were relevant for you to feel better in dealing with body stress patients? please choose your top 3 elements!	checkbox, Required<br>1 b10 1__ Better knowledge of diagnostics<br>2 b10 2__ More self-confidence in terms of my competence<br>3 b10 3__ That I could offer the patient something, e.g. physiotherapy<br>4 b10 4__ A clear algorithm that I can stick to<br>5 b10 5__ Tips and tricks for conducting conversations<br>6 b10 6__ The exchange with colleagues on the topic<br>7 b10 7__ Other<br><br>Custom alignment: LV<br>Field annotation: @MAXCHECKED=3
24	[b10_other]<br><br>Show the field ONLY if:<br>[b10(7)] = '1'		Please specify:	notes, Required<br>Custom alignment: LH
25	[b11]		Section Header:<br>17) How often do you provide education on the topic of the autonomic nervous system and body stress, even independently of inclusion in the project?	radio, Required<br>1 never<br>2 1x per week<br>3 2-4x per week<br>4 5-10x per week<br>5 more than 10 times a week<br><br>Custom alignment: LV
26	[b12]		18) How often do you use the (educational) materials for this?	radio, Required<br>1 never<br>2 1x per week<br>3 2-4x per week<br>4 5-10x per week<br>5 more than 10 times a week<br>Custom alignment: LV
27	[b13a]		Section Header: *19) Which of the materials do you use and how often?*<br>The diagram of the autonomic nervous system	radio (Matrix), Required<br>1 never<br>2 rare<br>3 mostly<br>4 always
28	[b13b]		The stress pattern	radio (Matrix), Required<br>1 never<br>2 rare<br>3 mostly<br>4 always
29	[b13c]		The movement scheme	radio (Matrix), Required<br>1 never<br>2 rare<br>3 mostly<br>4 always
30	[b13d]		PRISM	radio (Matrix), Required<br>1 never<br>2 rare<br>3 mostly<br>4 always
31	[b13e]		20) What materials are you missing?	notes<br>Custom alignment: LH

32	[b14]	Section Header:<br><br>21) What would help you feel better when dealing with body-stress patients? please choose your top 3 items!	checkbox, Required<br>1 | b14 1__ | Conversation management courses and techniques<br>2 | b14 2__ | Specific suggestions for wording - how do I address it?<br>3 | b14 3__ | Simply more routine<br>4 | b14 4__ | More practical access to the materials so I don't have to search for a long time<br>5 | b14 5__ | Regular exchange with colleagues<br>6 | b14 6__ | Professional supervision and mentoring<br>7 | b14 7__ | More information material for patients so that they can take something with them<br>8 | b14 8__ | A video for patients in which the topic is also explained visually<br>9 | b14 9__ | Other<br><br>Custom alignment: LV<br>Field annotation: @MAXCHECKED=3
33	[b14_other]<br><br>Show the field ONLY if:<br>[b14(9)] = '1'	Please specify:	notes, Required<br>Custom alignment: LH
34	[b15]	22) In your opinion, what was the most likely reason that in cases where you tried to the issue of body stress, it was not to establish a conversation or the patient no longer showed up for follow-up appointments?	notes, Required<br>Custom alignment: LH
35	[b16]	Section Header: *Interprofessional collaboration*<br><br>23) How often do you prescribe physiotherapy for patients with physical stress symptoms?	radio, Required<br>1 | frequently<br>2 | occasionally<br>3 | never<br><br>Custom alignment: LV
36	[c1]	24) When you think about the last 3 months, how good is your experience of working with physiotherapists? 0 = very poor; 10 = very good	radio, Required<br>0 | 0<br>1 | 1<br>2 | 2<br>3 | 3<br>4 | 4<br>5 | 5<br>6 | 6<br>7 | 7<br>8 | 8<br>9 | 9<br>10 | 10<br><br>Custom alignment: LH
37	[c3]	25) If you think back over the last 3 months, how often have you received feedback from the physiotherapists treating patients for whom you prescribed physiotherapy on the progress of their therapy?	radio, Required<br>1 | frequently<br>2 | occasionally<br>3 | never<br><br>Custom alignment: LV
38	[d1]	Section Header: *Assessment of acceptance and practicability of the body-stress care model for the future*<br><br>26) I will continue to the model in my consultation hours.0 = Does not apply at all; 10 = Fully applies	radio, Required<br>0 | 0<br>1 | 1<br>2 | 2<br>3 | 3<br>4 | 4<br>5 | 5<br>6 | 6<br>7 | 7<br>8 | 8<br>9 | 9<br>10 | 10<br><br>Custom alignment: LH

	39	[d2]	27) I would recommend the use of the model to friends and family doctor colleagues.0 = Does not apply at all; 10 = Fully applies	radio, Required<br>0 0<br>1 1<br>2 2<br>3 3<br>4 4<br>5 5<br>6 6<br>7 7<br>8 8<br>9 9<br>10 10<br><br>Custom alignment: LH
	40	[d3]	28) My patients' reactions to the following measures were particularly positive:(multiple answers possible)	checkbox, Required<br>1 d3 1__ The doctor-patient discussion on the subject of body stress itself<br>2 d3 2__ Education using the materials<br>3 d3 3__ Group coaching<br>4 d3 4__ Physiotherapy<br>5 d3 5__ The use of PRISM<br>6 d3 6__ Sleep therapy<br><br>Custom alignment: LV
	41	[text2]	Section Header: *Survey completed*<br>Thank you for answering the questions, please click on the "Submit" button to complete the questionnaire.	descriptive
	42	[gp_followup_com plete]	Section Header: *Form Status*<br>Complete?	dropdown<br>0 Incomplete<br>1 Unverified<br>2 Complete

4. Survey to physiotherapists

Languages	
ID	Display Name
en	German (default)

	C	Variable / Field Name	Field Label<br>***Field Note***	**Field Attributes (Field Type, Validation, Choices, Calculations, etc.)**
**Instrument: Pre-treatment survey** Active languages - Data Entry: en| Survey: en				**Enabled as survey**
	1	[record_id]	Record ID	text
	2	[begin]	Please click on the "Next page" button to start filling in the form.	descriptive, Required
	3	[p1]	Section Header:<br>Are you one of the physiotherapists involved in the project?	yesno, Required<br>1 Yes<br>0 No<br>Custom alignment: LV
	4	[p3]	Section Heade *t: Socio-demographic data*<br>Your year	text (integer, Min: 1 900, Max: 2022), Required<br>Custom alignment: RH<br>Field Annotation: @CHARLIMIT=4 AND @PLACEHOLDER='e.g. J 979'
	5	[p4]	Gender	radio, Required<br>1 female<br>2 male<br>3 diverse
	6	[p5]	Experience as a physiotherapist	text (integer, Min: 1, Max: 99), Required<br>Custom alignment: RH<br>Field annotation: @PLACEHOLDER='years'
	7	[p6]	Workload	text (integer, Min: 0, Max: 100), Required<br>Custom alignment: RH<br>Field annotation: @PLACEHOLDER='percent'
	8	[p7]	Section Header: *Assessment of psycho-samatic complaints*<br>How interested are you in the psychosocial aspects of physiotherapy?	radio, Required<br>1 little<br>2 medium<br>3 strong
	9	[p8]	In how many of the patients you have treated in the last week do you suspect a psychological cause of their symptoms7?	radio, Required<br>1 With none/none<br>2 1 to 5<br>3 6 to 1 0<br>4 For more than 1 0
	10	[p9]	Senion Header:<br>On a scale from 0 (very uncertain) to 100 (very certain), how confident do you feel when assessing patients for whom you suspect a "psycho-somatic" co-cause?	slider (number, Min: 0, Max: 100), Required Slider labels: very uncertain , very certain Custom alignment: RH
	11	[p10]	On a scale from 0 (very uncertain) to 1 00 (very certain), how confident do you feel in treating patients for whom you also suspect a "psycho-somatic" cause of the complaints?	slider (number, Min: 0, Max: 100), Required<br>Slider labels: very uncertain, very certain<br>Custom alignment: RN
	12	[p11]	Section Header:<br>How strong is your motivation to get involved in the treatment of "psycho-somatic" patients?	slider (number, Min: 0, Max: 100), Required Slider labels: very small, very large Custom alignment: RN
	13	[p12]	Do you need training in dealing with "psychosomatic" patients?	yesno, Required<br>1 Yes<br>0 No<br>Custom alignment: RH

Instruments	
**Instrument**	**Form Name**

Languages	
**ID**	**Display Name**
en	German **(default)**

	#	Variable / Field Name	Field Label<br>*Field Note*	Field Attributes (Field Type, Validation, Choices, Calculations, etc.)
**Instrument: Post-treatment survey**<br>Active languages - Data Entry: de | Survey: de				**Enabled as survey**
	1	[record_id]	Record ID	text
	2	[begin]	Please click on the "Next page" button to start filling in the form.	descriptive, Required
	3	[a1]	Section Header:<br>1) Are you one of the physiotherapists involved in the project?	yesno, Required<br>1 Yes<br>0 No<br>Custom alignment: LV
	4	[a2]	Section Header: *Socio-demographic information*<br>2) Your year	text (integer, Min: 1900, Max: 2022), Required Custom alignment: RH<br>Field Annotation: @CHARLIMIT=4 AND @PLACEHOLDER='e.g. 1979'
	5	[a3]	3) Gender	radio, Required<br>1 female<br>2 male<br>3 diverse
	6	[a4]	4) Experience as a physiotherapist	text (integer, Min: 1, Max: 99), Required<br>Custom alignment: RH<br>Field annotation: @PLACEHOLDER='years'
	7	[a5]	5) Workload	text (integer, Min: 0, Max: 100), Required<br>Custom alignment: RH<br>Field annotation: @PLACEHOLDER='percent'
	8	[a6]	Section Header: *Socio-demographic data (continued)*<br>6) I have taken part in training courses on the subject of body stress.	yesno, Required<br>1 Yes<br>0 No
	9	[a7]	7) I already took part in the first survey in January 2023.	yesno, Required<br>1 Yes<br>0 No
	10	[a8]	8) I looked after about this many patients in the project:	radio, Required<br>1 none<br>2 1-5<br>3 6-10<br>4 more than 10
	11	[text_01]	Section Header: *Assessment of competence in relation to the treatment of body stress patients*<br>In the following questions, we would like to record the effects of the body stress project for you.	descriptive
	12	[b1]	9) I now feel more confident in dealing with patients with body stress symptoms.0= Not at all; 10= Clearly	radio, Required<br>0 0<br>1 1<br>2 2<br>3 3<br>4 4<br>5 5<br>6 6

				7 7<br>8 8<br>9 9<br>10 10<br><br>Custom alignment: LH
	13	[b2]	10) Which elements were relevant for you to feel better in dealing with body stress patients? Please choose your top 3 elements!	checkbox, Required<br>1 b2___1 Body stress syndrome diagnosed and listed on the prescription<br>2 b2___2 Better knowledge of the disease<br>3 b2___3 More self-confidence terms of my competence<br>4 b2___4 Better coordination of work with general practitioners<br>5 b2___5 A clear algorithm that I can stick to<br>6 b2___6 Tips and tricks for conducting conversations<br>7 b2___7 The exchange with colleagues on the topic<br>8 b2___8 Other<br><br>Custom alignment: LV<br>Field annotation: @MAXCHECKED=3
	14	[b2_text]<br><br>Show the field ONLY if:<br>[b2(8)] = '1'	Please specify:	notes, Required<br>Custom alignment: LH
	15	[b3]	11) What is the percentage of patients in whom you suspect physical stress to be the cause of their complaints?	text (integer, Min: 0, Max: 100), Required<br>Field annotation: @PLACEHOLDER='In percent'
	16	[b4a]	Section Header: *12) You probably do not address psychosomatic complaints in every treatment, even if you recognize them. To what extent are the following reasons for this? (0=not at all; 10=absolutely)*<br><br>I still don't feel professionally competent in the therapy of body stress patients.	radio (Matrix), Required<br>0 0<br>1 1<br>2 2<br>3 3<br>4 4<br>5 5<br>6 6<br>7 7<br>8 8<br>9 9<br>10 10
	17	[b4b]	I don't manage to bridge the gap in the conversation and address the issue.	radio (Matrix), Required<br>0 0<br>1 1<br>2 2<br>3 3<br>4 4<br>5 5<br>6 6<br>7 7<br>8 8<br>9 9<br>10 10
	18	[b4c]	The care of body-stress patients is time-consuming. During my regular	radio (Matrix), Required<br>0 0

			physiotherapy work, there is no time to deal with psychosomatic problems.	1 1<br>2 2<br>3 3<br>4 4<br>5 5<br>6 6<br>7 7<br>8 8<br>9 9<br>10 10
	19	[b5]	Section Header:<br>13) How often do you now consciously address the topic of the autonomic nervous system and body stress, even independently of the project?	radio, Required<br>1 never<br>2 1-2x per week<br>3 more than 3x per week
	20	[b6]	14) What would help you feel better when dealing with body stress patients? Please your top 3 elements!	checkbox, Required<br>1 b6 ___1 Conversation management courses techniques<br>2 b6 ___2 Specific suggestions for wording - how do I address it?<br>3 b6 ___3 Simply more routine<br>4 b6 ___4 More practical access to the materials so I don't have to search for a long time<br>5 b6 ___5 Regular exchange with colleagues<br>6 b6 ___6 Professional support and supervision by mentors<br>7 b6 ___7 More information material for patients so that they can take something with them<br>8 b6 ___8 A video for patients in which the topic is also explained visually<br>9 b6 ___9 Closer cooperation with general practitioners<br>10 b6 ___10 Specialist psychiatric-psychosomatic training<br>11 b6 ___11 Other<br><br>Custom alignment: LV<br>Field annotation: @MAXCHECKED=3
	21	[b6_text]<br><br>Show the field ONLY if:<br>[b6(11)] = '1'	Please specify:<br><br>Section Header:	notes, Required<br>Custom alignment: LH
	22	[b7]	15) How much of your working time would you like to spend with body-stress patients?	text (Integer, Min: 0, Max: 100), Required<br>Field annotation: @PLACEHOLDER='In percent'
	23	[b8]	16) Would you recommend working with body-stress patients to your colleagues?	yesno, Required<br>1 Yes<br>0 No
	24	[b8_text]<br><br>Show the field ONLY if:<br>[b8] = '0'	Why not?<br><br>Section Header: *Cooperation with doctors*	notes, Required<br>Custom alignment: LH
	25	[c1]	17) To what extent has the collaboration with the GPs changed as a result of the project?	radio, Required<br>1 There is less exchange than before<br>2 Nothing has changed<br>3 There is more exchange than before

				Custom alignment: LV
	26	[c2]	18) How, or with what measures, could cooperation with general practitioners for the treatment of psycho-somatic patients improved?	notes<br>Custom alignment: LH
	27	[c3]	19) In your opinion, what are the main barriers that hinder cooperation between doctors and physiotherapists?	notes<br>Custom alignment: LH
	28	[end]	Section Header: *Survey completed*<br>Thank you for answering the questions, please click on the "Submit" button to complete the questionnaire.	descriptive
	29	[physiotherapie_followup_complete]	Section Header: *Form Status*<br>Complete?	dropdown<br>0 Incomplete<br>1 Unverified<br>2 Complete
