# Supplementary material for: Factors influencing appropriate vestibular care: An interview study with general practitioners and patients
Source: Eur J Gen Pract. 2025 Dec 16;31(1):2600144. doi: 10.1080/13814788.2025.2600144 (PMC12710262; doi:10.1080/13814788.2025.2600144)
Supplement: Supplemental Material [file IGEN_A_2600144_SM5512.zip › suppl_data/ejgp-2025-0201-File008.docx]

**Supplementary table 1. Topic list for general practitioners.**

| Subject | Example question |
| --- | --- |
| Experiences with patients with vestibular symptoms | Main question   - Can you tell about the last time that a patient with vestibular symptoms came for a consultation? How did that go?   Other questions   - What is easy or difficult for you about treating patients with vestibular symptoms? - What would make the management of patients with vestibular symptoms easier? - How confident are you about the management of patients with vestibular symptoms? |
| Experiences with anti-vertigo drugs | Main question   - Are you familiar with anti-vertigo drugs? What are your experiences with these drugs?   Other questions   - What were reasons to prescribe anti-vertigo drugs? - To what extent did the patient play a role in the prescription of anti-vertigo drugs? |
| Domain: capability | Main questions   - To what extent are you familiar with the current guidelines for the management of vestibular symptoms? - To what extent do you feel competent to treat patients with vestibular symptoms?   Other questions   - What is your opinion about the current guidelines for the management of vestibular symptoms? - To what extent did you learn about vestibular symptoms? - Have you heard of vestibular rehabilitation? What are your experiences with vestibular rehabilitation? - Would you apply vestibular rehabilitation? Why (not)? - What yould help you to apply vestibular rehabilitation? - What would help you to stop prescribing anti-vertigo drugs? |
| Domain: opportunity | Main questions   - How do your colleagues treat patients with vestibular symptoms? - What do you think the chances are that you stop prescribing anti-vertigo drugs? Why?   Other questions   - What do you think the chances are that you apply vestibular rehabilitation? Why? - What makes it easy or difficult to stop prescribing anti-vertigo drugs? - What makes it easy or difficult to apply vestibular rehabilitation? - To what extent have you learned to treat patients with vestibular symptoms during your residency? |
| Domein: motivation | Main questions   - What are advantages and disadvantages of stopping with prescribing anti-vertigo drugs? - What role do you see for yourself in the treatment of patients with vestibular symptoms?   Other questions   - What would motivate you to stop prescribing anti-vertigo drugs? - What would motivate you to apply vestibular rehabilitation? |
| Implementation strategy | Main question   - How do you think we can raise awareness of vestibular rehabilitation?   Other questions   - How do you think we can decrease the number of prescriptions for anti-vertigo drugs? |

**Supplementary table 2. Topic list for patients with vestibular symptoms.**

| Subject | Example question |
| --- | --- |
| Experiences with (treatments for) vestibular symptoms | Main question   - Can you tell about the last time that you visited the general practitioner because of vestibular symptoms? How did that go?   Other questions   - How has the general practitioner treated the vestibular symptoms? - To what extent did you have an influence on the treatment? |
| Experiences with anti-vertigo drugs | Main question   - Are you familiar with anti-vertigo drugs? What are your experiences with these drugs?   Other questions   - How did the treatment with anti-vertigo drugs come into being? - To what extent did you play a role in the prescription of anti-vertigo drugs? |
| Domain: capability | Main questions   - To what extent were you familiar with anti-vertigo drugs before you started using them? - To what extent did you look up information about treatments for vestibular symptoms?   Other questions   - What would help you to stop using anti-vertigo drugs? - Have you heard of vestibular rehabilitation? - What would help you to use vestibular rehabilitation? |
| Domain: opportunity | Main questions   - What would be easy or difficult about stopping with using anti-vertigo drugs?   Other questions   - What do you think the chances are that you would stop using anti-vertigo drugs? - What do you think the chances are that you will start using vestibular rehabilitation? - To what extent have you had contact with other patients with vestibular symptoms? Do they also use anti-vertigo drugs? |
| Domein: motivation | Main questions   - What are advantages and disadvantages of stopping with using anti-vertigo drugs? - What would motivate you to stop using anti-vertigo drugs?   Other questions   - What would motivate you to use vestibular rehabilitation? - To what extent do you think anti-vertigo drugs help you with the complaints? - To what extent do you think vestibular rehabilitation (will) help you with the complaints? |
| Implementation strategy | Main question   - How do you think we can raise awareness of vestibular rehabilitation?   Other questions   - How do you think we can decrease the number of prescriptions for anti-vertigo drugs? |
