# Supplementary figures and images for: Factors influencing appropriate vestibular care: An interview study with general practitioners and patients
Source: Eur J Gen Pract. 2025 Dec 16;31(1):2600144. doi: 10.1080/13814788.2025.2600144 (PMC12710262; doi:10.1080/13814788.2025.2600144)

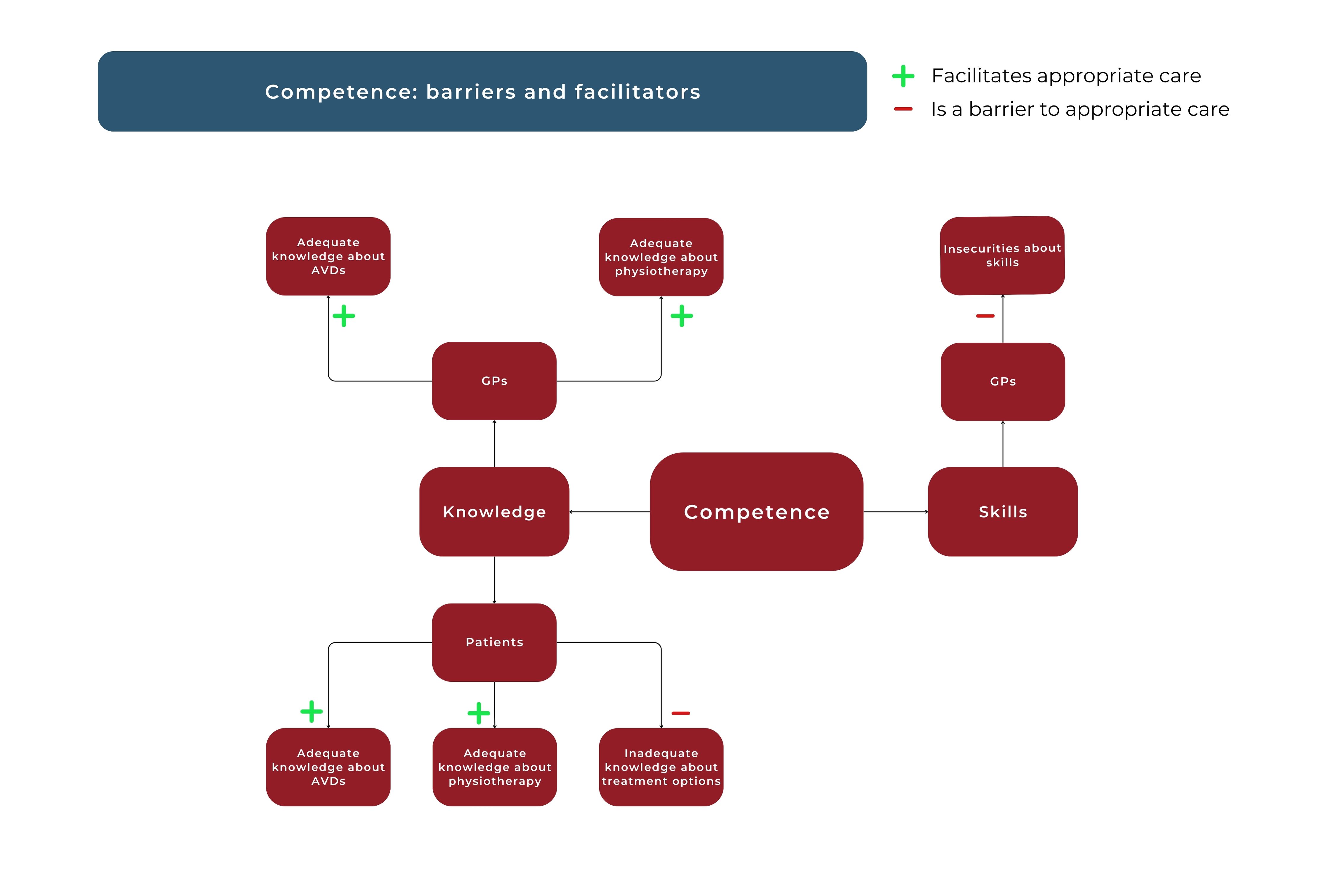

Supplement: Supplemental Material [file IGEN_A_2600144_SM5512.zip › suppl_data/ejgp-2025-0201-File004.jpg]

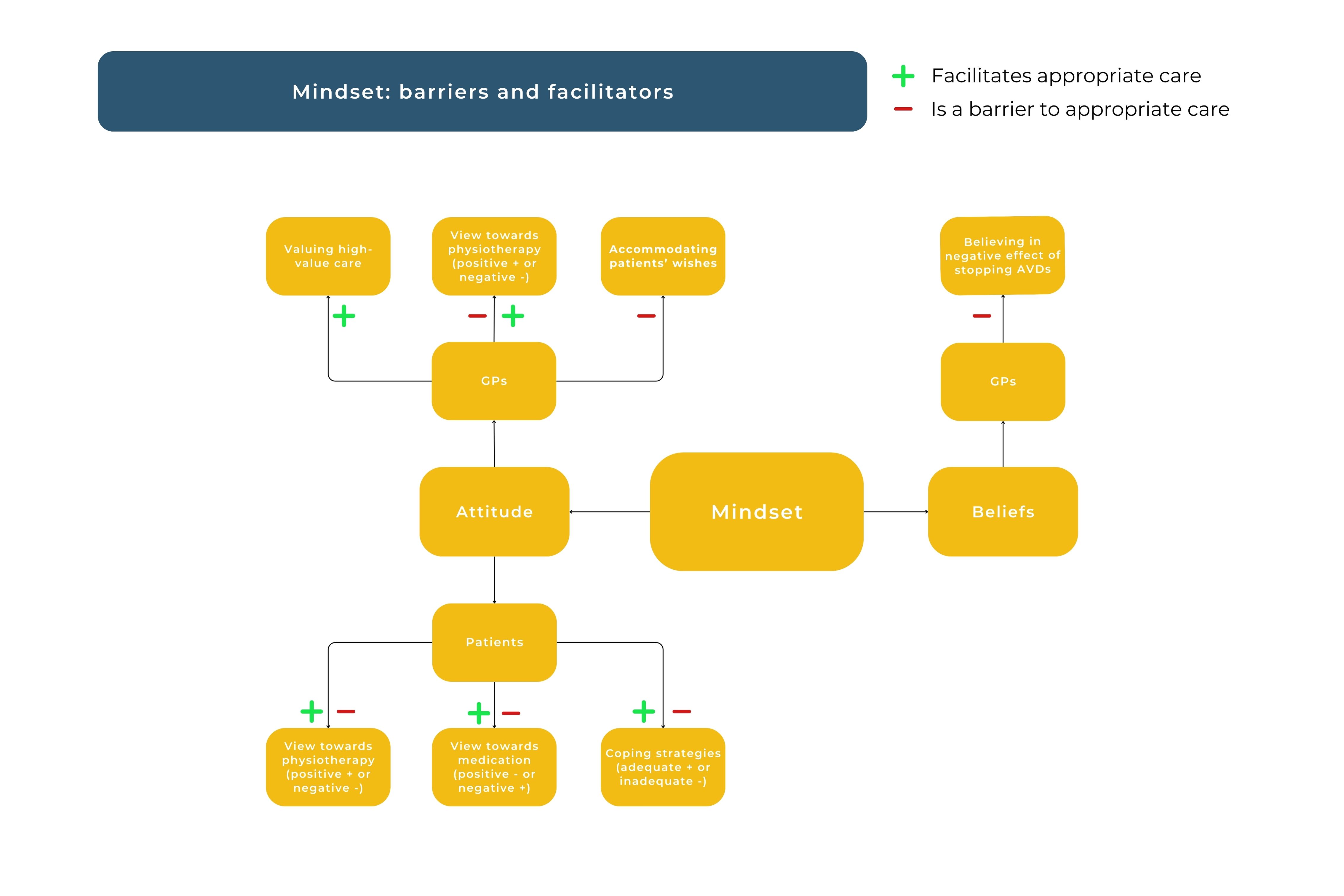

Supplement: Supplemental Material [file IGEN_A_2600144_SM5512.zip › suppl_data/ejgp-2025-0201-File005.jpg]

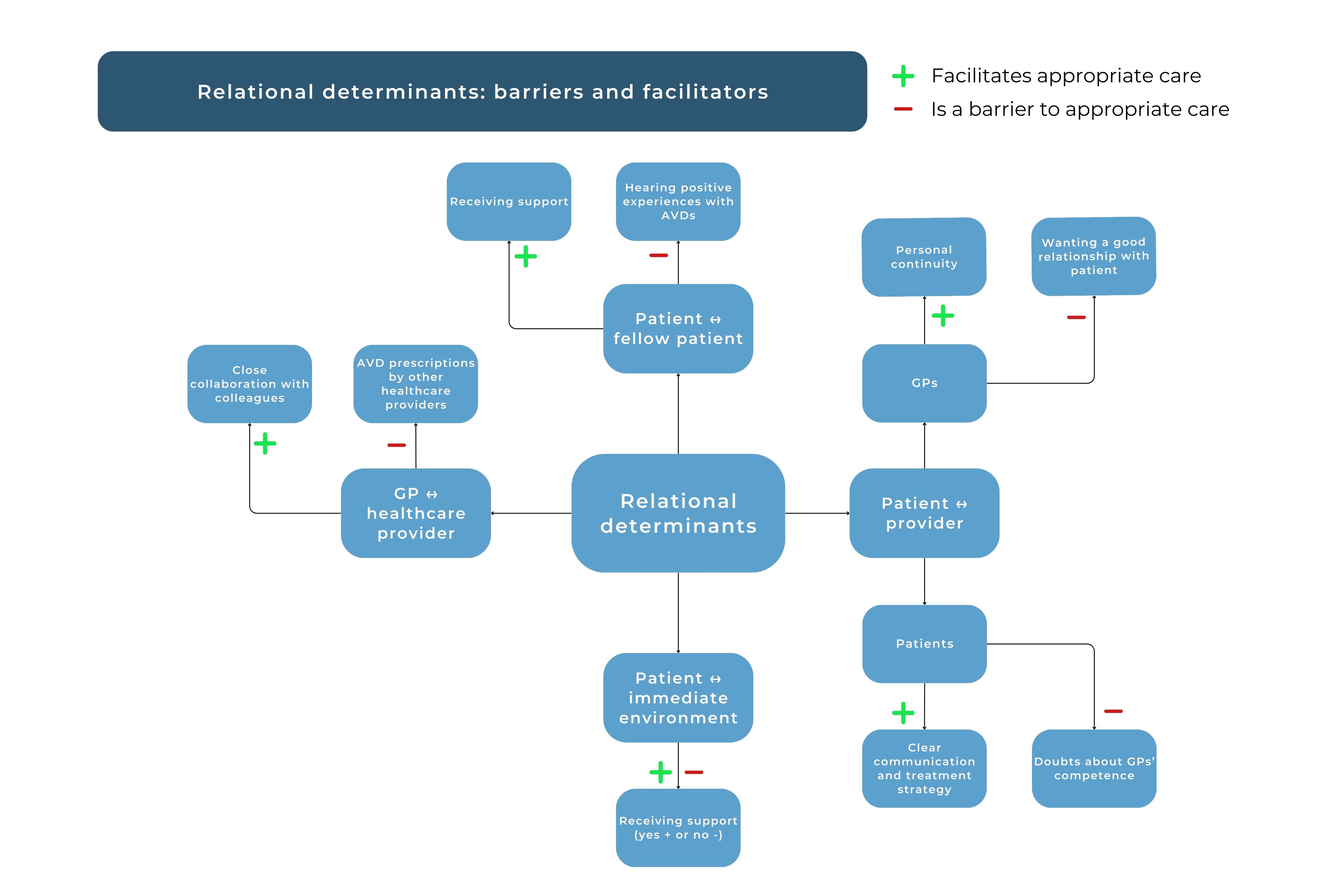

Supplement: Supplemental Material [file IGEN_A_2600144_SM5512.zip › suppl_data/ejgp-2025-0201-File006.jpg]

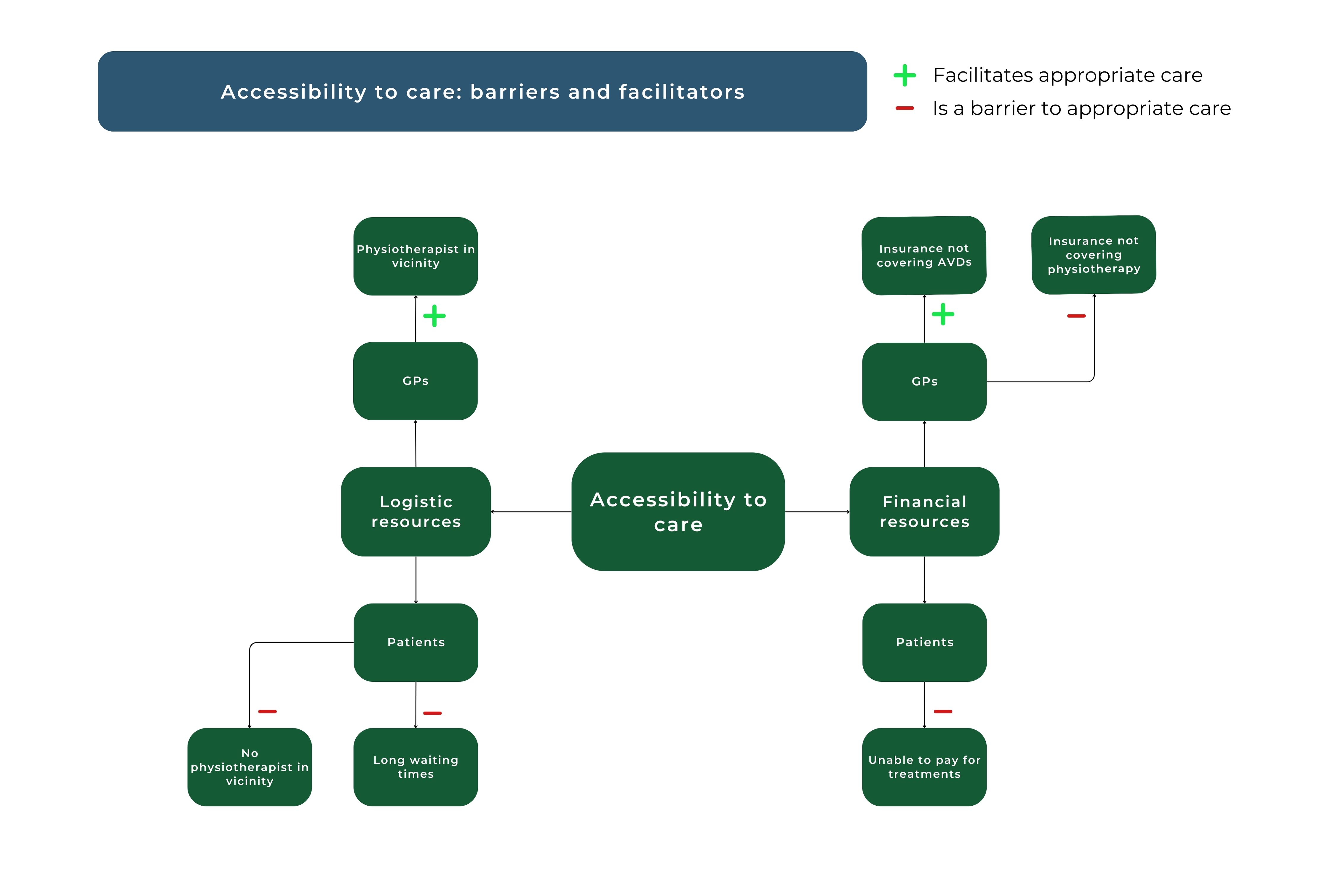

Supplement: Supplemental Material [file IGEN_A_2600144_SM5512.zip › suppl_data/ejgp-2025-0201-File007.jpg]
